# Dynamical models of mutated chronic myelogenous leukemia cells for a post-*imatinib* treatment scenario: Response to *dasatinib* or *nilotinib* therapy

**DOI:** 10.1371/journal.pone.0179700

**Published:** 2017-07-05

**Authors:** Clemens Woywod, Franz X. Gruber, Richard A. Engh, Tor Flå

**Affiliations:** 1 Centre for Theoretical and Computational Chemistry, Chemistry Department, University of Tromsø - The Arctic University of Norway, N-9037 Tromsø, Norway; 2 NORSTRUCT, Chemistry Department, University of Tromsø - The Arctic University of Norway, N-9037 Tromsø, Norway; 3 Mathematics Department, University of Tromsø - The Arctic University of Norway, N-9037 Tromsø, Norway; University of California Irvine, UNITED STATES

## Abstract

Targeted inhibition of the oncogenic BCR-ABL1 fusion protein using the ABL1 tyrosine kinase inhibitor *imatinib* has become standard therapy for chronic myelogenous leukemia (CML), with most patients reaching total and durable remission. However, a significant fraction of patients develop resistance, commonly due to mutated ABL1 kinase domains. This motivated development of second-generation drugs with broadened or altered protein kinase selectivity profiles, including *dasatinib* and *nilotinib*. *Imatinib*-resistant patients undergoing treatment with second-line drugs typically develop resistance to them, but dynamic and clonal properties of this response differ. Shared, however, is the observation of clonal competition, reflected in patterns of successive dominance of individual clones. We present three deterministic mathematical models to study the origins of clinically observed dynamics. Each model is a system of coupled first-order differential equations, considering populations of three mutated active stem cell strains and three associated pools of differentiated cells; two models allow for activation of quiescent stem cells. Each approach is distinguished by the way proliferation rates of the primary stem cell reservoir are modulated. Previous studies have concentrated on simulating the response of wild-type leukemic cells to *imatinib* administration; our focus is on modelling the time dependence of *imatinib*-resistant clones upon subsequent exposure to *dasatinib* or *nilotinib*. Performance of the three computational schemes to reproduce selected CML patient profiles is assessed. While some simple cases can be approximated by a basic design that does not invoke quiescence, others are more complex and require involvement of non-cycling stem cells for reproduction. We implement a new feedback mechanism for regulation of coupling between cycling and non-cycling stem cell reservoirs that depends on total cell populations. A bifurcation landscape analysis is also performed for solutions to the basic ansatz. Computational models reproducing patient data illustrate potential dynamic mechanisms that may guide optimization of therapy of drug resistant CML.

## Introduction

With the discovery of tyrosine kinase inhibitor (TKI) drugs, a diagnosis of early stage chronic myelogenous leukemia (CML) became associated with a prognosis of normal life expectancy [1]. The success of the TKI drugs for CML may be attributed to the fact that nearly all CML cancers are caused by a single molecular lesion, creating a uniquely homogeneous cancer drug target, along with the relative genetic stability of the early stage oncogenic hematopoetic myeloid cells. Untreated CML advances to the accelerated phase and finally blast crisis, with increased genetic instability, total tumor burden, and characteristics of acute leukemia. Despite the success of TKI drugs in early stage CML, late diagnosis—and progression of the disease in some patients despite treatment—may involve resistance to TKI therapy. Current drug discovery efforts aim to identify new TKI inhibitors that can replace drugs rendered ineffective by resistance mutations, and to forestall or even prevent TKI drug resistance. These efforts are aided by increasing understanding of the molecular processes that underlie the dynamics of clinical resistance, which in turn should ultimately enable predictive modelling of optimized disease therapy.

Despite great progress in collecting disease relevant data [2], the origins and complexities of leukemias within the hematopoietic cell hierarchy are not well understood [3]. In the case of CML [4], the reciprocal t(9;22)(q34;q11) translocation that creates the “Philadelphia chromosome” and its BCR-ABL1 fusion protein occurs in hematopoietic stem cells [5]. The fusion removes the regulatory domains from the ABL1 kinase domain, rendering it constitutively active, although it retains the ability to adopt both active and inactive conformations, related to folding geometries of the so-called “DFG” segment that forms part of the ATP binding site. The first of the successful TKI drugs against CML, *imatinib*, inhibits the BCR-ABL1 tyrosine kinase by occupying the ATP-binding site, locking the protein in an inactive conformation, switching off a variety of the downstream signalling events [6]. *Imatinib* both inhibits proliferation and increases apoptosis of actively proliferating wild-type Ph^+^(Philadelphia chromosome-positive) progenitor cells, but the effect on cycling and non-cycling CML stem cells is a focus of current research [7].

Despite the successes of TKI therapy of CML, a significant fraction of patients develop resistance to treatment. In approximately half of these cases, resistance arises from mutations of the ABL1 kinase domain. This was first observed for *imatinib* therapy, whereby the resistant cancer in some cases possessed multiple and competing resistant clones. The observation of *imatinib* resistance led to the development of alternative TKI drugs against CML; *dasatinib*, *nilotinib*, and *ponatinib* have been approved for clinical use [8]. While these have not replaced *imatinib* for first-line therapy, they can be used for *imatinib*-resistant CML, but may also be rendered ineffective by Ph^+^ cell mutation [9]. A key issue with respect to permanent eradication of Ph^+^ cells (and thereby to cure CML) is the accessibility and effect of TKI therapy on Ph^+^ stem cells [10–16]. Recent bone marrow studies have shown the sensitivity of residual wild-type and mutant Ph^+^ cancer stem cells during *imatinib* and *dasatinib* treatment, indicating that in particular *dasatinib* therapy can rapidly eliminate leukemic stem cells *in vivo* [17].

Four mechanisms have been proposed to explain the continued presence of cycling wild-type Ph^+^ stem cells despite treatment: (i) Proliferating stem cells are suppressed by *imatinib* but quiescent cells are not. (ii) *Imatinib* is eliminated from the cytoplasm of proliferating CML stem cells. (iii) Cycling stem cells have a higher production rate of the BCR-ABL1 protein compared to progeny cells. (iv) The immune system responds to progeny cells, but not to Ph^+^ stem cells.

Clinical data and knowledge of CML disease mechanisms have supported a variety of efforts to model CML and resistance dynamics, ultimately with the aim of optimizing therapy. Essential features of the evolution of both normal and leukemic cells are well understood. However, differential effects of TKI inhibitors are less well understood, in particular at the stem cell level; models illustrate and may help clarify the effects of different therapies on stem cell proliferation, differentiation, and apoptosis rates [18]. Several approaches have been used to model the persistence of the wild-type leukemia stem cells during *imatinib* therapy, most significantly differing with respect to the treatment of quiescence.

Before discussing the different computational concepts, a remark on nomenclature: In Refs. [19–21], stem cell growth environments (bone marrow niches supporting either cycling or non-cycling stem cells) are also referred to as “signalling contexts”, while Refs. [22, 23] use the term “compartments”. For clarity, we define the expression “compartment” to mean the individual layers of the differentiation hierarchy of the haematopoietic system as proposed e.g. in Refs. [15, 24]. Accordingly, the stem cell compartment is composed of two growth environments: active and quiescent.

Michor *et al.* first described a model that features both normal and leukemic versions of cycling stem cells, progenitors, differentiated and terminally differentiated cells [15]. The model distinguished quiescent from proliferating stem cells, but did not include sensitivity of the stem cell compartment to *imatinib* treatment. The biphasic decay of BCR-ABL1 transcripts measured in blood following *imatinib* treatment was thereby interpreted as a rapid initial decay of differentiated leukemic cells succeeded by a slower decay of leukemic progenitors.

Roeder *et al.* [20] use a stochastic approach (“agent based model” (ABM) [21]) that considers stem cells to switch between activated and quiescent states, assuming that *imatinib* affects only the activated stem cells. This model attributes the clinically observed biphasic decline of BCR-ABL1 transcript levels to the faster effect on activated stem cells and the slower repopulation from the quiescent pool.

Because switching between active and quiescent states implies some form of signalling via stem cell niche interactions, this view allows for competition between mutant Ph^+^ stem cell clones that may possess varying responses to the niche environment. If the clones are differentially sensitive to TKIs, therapy may alter the overall composition of the stem cell pool such that clones best suited to niche competition under treatment come to dominate. Thus, complete modelling of the clinical effects of TKI therapy must take into account multiple interdependent factors: enzymatic activities of BCR-ABL1 variants, relative substrate selectivities, proliferation vs. differentiation vs. quiescence transition rates, and effects of non-ABL1 tyrosine kinase inhibition, to name a few [18].

Subsequent studies have refined or extended these early approaches. Komarova and Wodarz [25] introduced a stochastic model that explicitly includes populations of both cycling and non-cycling stem cells in order to explain biphasic decay of wild-type CML cell populations upon treatment with *imatinib*. Levy *et al.* added immune system response terms [26, 27]. Michor *et al.* included the quiescent state of stem cells explicitly in a deterministic model [28]. Roeder *et al.* [19] and Doumic-Jauffret *et al.* [22] approximated the earlier stochastic model with a faster deterministic partial differential equation (PDE) model.

Unlike these investigations, which are primarily devoted to the study of *imatinib* therapy, the focus of the present modelling study is on the time development of CML cells in the regime of exposure to *dasatinib* or *nilotinib*, after suspension of *imatinib* therapy due to resistance.

In Ref. [29], the dynamical model described in Ref. [28] is applied to simulate the response of wild-type CML cells to treatment with *dasatinib*, *nilotinib* and high-dose *imatinib*. The dynamics of resistant CML mutants is not considered in Ref. [29]. Ref. [30] presents a stochastic model for the evolution of CML mutations resistant to TKIs before therapy is initialized.

We describe three versions of a deterministic approach to the description of the dynamics of CML stem and differentiated cells. The models differ with respect to the role of the niche environment on stem cell proliferation via activation of quiescent stem cells. The simulations of the time evolution of three strains of each type of stem and differentiated cancer cells are compared to recently acquired clinical data.

## Materials and methods

### (2.1) General

We build on existing models [15, 28, 31–34], and introduce negative feedback signals to study the clonal competition phenomena as seen in clinical data described above. Our goal is to investigate the dynamics of the populations of drug resistant clones as observed in CML patients who were treated by *dasatinib* or *nilotinib* after their disease became resistant to *imatinib* [35–42]. Certain patterns are especially noteworthy, such as the rapid emergence of one resistant clone, to be overtaken by another over the course of treatment, or relapse phenomena. Table 1 provides an overview of typical curve progressions observed in the time evolutions of differentiated cell populations of individual *imatinib*-resistant clones as reported in Ref. [41].

**Table 1:** Schematic classification of the time evolution of individual CML clones as observed in the fourteen patient profiles compiled in Fig 2 of Ref. [41]. The clonal time courses can be divided into ‘classes’ based on their common abstracted characteristics. The resulting eight classes of time courses are ordered according to increasing complexity.

Description of clone development	CML clone	Patient profile(s)
1-phasic decay	M351T	D13, N16, N25, N34, N35
	Y253H	D7, D11
1-phasic growth	E255K	N35
	E255V	N34
	F311I	N25
	T315I	D11
2-phasic decay	F317L	N34, N35
2-phasic growth	V299L_1	D14
	V299L_2	D14
3-phasic decay	Y253H	D14, D15
Decay → minimum → growth	F359V	N40
	T315I	D7
Growth → maximum → decay	E255V	D8, N16
	F317L	D7, D11, D13, D14
	T315I	D15, N5, N22
	Y253H	N40
Growth → maximum (local / global) → decay → minimum (local / global) → growth	E255K	D8, N33
	T315I	N35
	Y253F	N33

The time-dependent populations of BCR-ABL1 stem and differentiated cell mutants *i* are given by *x*_*si*_(*t*) and *x*_*di*_(*t*), respectively; the total sets of stem and differentiated cell populations are represented by vectors **x**_*s*_(*t*) and **x**_*d*_(*t*) (i.e. **x**_*k*_(*t*) ≔ (*x*_*k*1_(*t*), *x*_*k*2_(*t*), …), *k* ∈ {*s*, *d*}). In the models we consider here, stem cell populations increase via symmetric division, while differentiated cell populations increase via asymmetric division of the parent stem cells. Following Ref. [31], we incorporate terms into the model to account for negative feedback control of these two growth mechanisms. Thereby, the (mutant specific) symmetric and asymmetric division rate constants *α*_*i*_ and *a*_*i*_, respectively, are multiplied by population dependent feedback or growth functions *ζ*_*i*_(**x**_*s*_(*t*)) and *p*(**x**_*d*_(*t*)) to represent signals or niche competition factors that limit growth and differentiation, respectively. We assume that the total populations of stem cells limit symmetric division via niche competition and possibly signalling, and that the total populations of differentiated cells limit asymmetric division via signalling.

Alternatively, a stem cell may divide symmetrically to yield two progenitor daughter cells [43]. This process, which implies death of the stem cell, will not be considered in the present study. For a discussion of the probabilities for differentiation pathways see e.g. Ref. [44].

We allow for clone-clone specific effects in stem cell niche competition, defining an array ***ω*** ≔ {*ω*_*ij*_} that represents the effect of clone *j* on clone *i* via a clone-specific threshhold *θ*_*si*_ such that negative feedback is switched on via an exponential factor when the weighted total distribution of competition effective mutants surpasses the threshhold (i.e. as ∑_*j*_ *ω*_*ij*_ *x*_*sj*_ > *θ*_*si*_).

For differentiated cells, we assume the simpler case whereby negative feedback is generated by signals representing the total population, without clone specificity. The cell populations also decay with rate constants *δ*_*i*_ and *d*_*i*_ for stem and differentiated cells, respectively.

Although the growth functions *ζ*_*i*_(**x**_*s*_(*t*)) and *p*(**x**_*d*_(*t*)) of stem and differentiated cells, respectively, are more accurately described by sigmoidal functions of the corresponding signals ∑_*j*_ *ω*_*ij*_ *x*_*sj*_ and ∑_*j*_ *x*_*dj*_ [28, 32–34], we will for convenience approximate the rapid variations of both functions in the vicinity of the thresholds *θ*_*ki*_ (*k* = *s*, *d*) by exponential functions. This choice of growth functions to switch off proliferation and differentiation in response to population levels is taken to follow existing literature [15, 31]. We also note that the molecular mechanisms for the feedbacks are not known, and, because the BCR-ABL1+ cells have escaped normal regulation, it is not clear that a steep switching mechanism should be assumed. The term “threshold” for *θ*_*ki*_ is therefore used in the sense of the so-called “e-folding threshold” [45] in the present study since an unique level, such as the inflection point of a sigmoidal curve, cannot be distinguished for exponential functions.

Thus, the basic differential equations for stem cell proliferation become:

$$\begin{array}{c} \frac{\text{d}x_{si}\left(t\right)}{\text{d}t}=\zeta_{i}\left(x_{s}\left(t\right)\right)\alpha_{i}x_{si}\left(t\right)-\delta_{i}x_{si}\left(t\right) \end{array}$$ (1a)

$$\begin{array}{c} \zeta_{i}\left(x_{s}\left(t\right)\right)≔\exp(-\sum_{j}\omega_{ij}x_{sj}\left(t\right)/\theta_{si}) \end{array}$$ (1b)

while those for differentiated cells are:

$$\begin{array}{c} \frac{\text{d}x_{di}\left(t\right)}{\text{d}t}=p\left(x_{d}\left(t\right)\right)\;a_{i}x_{si}\left(t\right)-d_{i}x_{di}\left(t\right) \end{array}$$ (2a)

$$\begin{array}{c} p\left(x_{d}\left(t\right)\right)≔\exp(-\sum_{j}x_{dj}\left(t\right)/\theta_{d}) \end{array}$$ (2b)

Other specific phenomena may be built into this basic model, and some modifications create the three models we analyse here. Mutation rates may be introduced, with corresponding effects on the relative populations.

We follow Ref. [23] and consider mutations only at the stem cell level. The rate term *r*_*i* → *j*_ describing the probability of mutations from clone *x*_*si*_(*t*) into *x*_*sj*_(*t*) per division of a cell of clone *x*_*si*_(*t*) is formulated according to:

$$\begin{array}{c} r_{i\to j}≔\nu_{j}\zeta_{i}\left(x_{s}\left(t\right)\right)\alpha_{i}x_{si}\left(t\right), \end{array}$$ (3)

where *ν*_*j*_ represents the mutation rate constant (see also definition of models A (Sec. 2.3), B (Sec. 2.4) and C (Sec. 2.5)).

More importantly, the stem cell pool may be distinguished into two growth environments to differentiate between quiescent (inactive, non-cycling) and proliferating (active, cycling) stem cells [15, 19–23, 25–28]. This becomes essential for reproducing aspects of patient data, in particular bi- or multiphasic decay patterns as well as recurrences of CML cell populations (see models B and C, results and discussion).

For the solution of the initial value problems we employ the *ode45* integrator (based on a Runge-Kutta (4, 5) algorithm [46]) as implemented in the *MATLAB* package [47] for the solution of non-stiff ordinary differential equations [48]. We tested also *MATLAB*s *ode15s* integrator (for stiff differential equations) and found results to be consistent with those obtained using *ode45*.

### (2.2) Empirical choices for model parameters

#### (2.2.1) Test simulations (qualitative) and patient simulations (quantitative)

The stem and differentiated cell mutants *x*_*si*_(*t*) and *x*_*di*_(*t*) represent the variants observed in patients that had acquired resistance mutations under *imatinib* treatment [35–41]; a major goal of this work is to investigate the underlying causes of their observed variations after initiation of new therapy. The patient data are not sufficient in number or quality to fit all parameters statistically. In the simulations of stem and differentiated cell dynamics performed for this study, we therefore differentiate between two types: (i) Test simulations that investigate the qualitative ability of the three models defined in Secs. 2.3, 2.4 and 2.5 to reproduce general patterns observed in the clinical cell population data presented in Refs. [40, 41]. In these simulations we follow the literature for additional information to compensate for the underdetermination of the model. (ii) Patient simulations of two selected clinical cases. The patient data profiles will generally be denoted PP# in this manuscript, referring to the numbering in Ref. [41]. We focus on PP14 and PP15, parameterizing models B and C to fit the experimental results. Tables 2 and 3 summarize the parameters used in the test and patient simulations of this study, respectively.

#### (2.2.2) Properties of clinical reference cohort

In selecting PP14 and PP15, we were interested in CML cell population evolutions which exhibit bi- or multiphasic decay patterns under second-line *dasatinib* or *nilotinib* administration because this type of response has been identified as a hallmark of the involvement of quiescent stem cells in the context of first-line treatment with *imatinib* [19, 25, 27, 28, 49].

Inspection of the PPs of Ref. [41] reveals that the reactions of the differentiated cell populations of clone Y253H to *dasatinib* therapy as recorded in PP14 and PP15 prove to be particularly suitable data samples for the desired multiphasic decay patterns. In addition, PP14 and PP15 are also representative of the total cohort of fourteen PPs in the sense that one (PP15) or three (PP14) additional *imatinib*-resistant strains with low cell populations at the beginning of second-line TKI application are emerging in the course of treatment.

A total of 59 resistant mutant clones have been identified from the *imatinib*-resistant patient data, 48 of which could be monitored during post-*imatinib* treatment [38, 40, 41] with *dasatinib* or *nilotinib*. The 10 amino acid substitutions corresponding to the nucleotide mutations (G250E, Y253(F,H), E255(K,V), V299L, T315I, F317L, M351T, F359V) alter the properties of the ABL1 kinase domain both with respect to drug binding and kinase activity, and so alter the best fit parameters for use in modelling the dynamics of the individual clones. For patients at this stage of drug resistant disease, the stem cells are overwhelmingly BCR-ABL1+, and the mutant forms are either detectable (as resistant mutations), pre-exist at low levels or may originate during second-line treatment. Model A (Secs. 2.3, 3.2) examines the kinetic requirements for the appearance of new mutants. Fourteen patients show dynamic properties of 1-4 resistant clones (cf. Figs 2a, 2b and 2c of Ref. [41]) over a 12-15 months treatment period with either *nilotinib* or *dasatinib*.

A classification of characteristic features of the time developments of the differentiated cell populations of CML clones as reported for the PPs of Ref. [41] is attempted in Table 1. The general patterns of dynamics that are of particular interest to reproduce with the models include the initial appearance of resistant clones, the apparent clonal competition (especially the rapid appearance of one clone initially with subsequent replacement by a second clone), bi- or multiphasic decline, and temporary stabilization or partial recovery of individual clones.

#### (2.2.3) Details of parameterization

If possible, we wish to draw conclusions with respect to persistence and sensitivities to drug administration, and to clonal competition, as a function of state of activity or differentiation. We implement our models usually to allow for three different variants of *x*_*si*_(*t*) and *x*_*di*_(*t*), only model C is reduced to a two clone system for one specific patient simulation (cf. Sec. 3.4.2). Corresponding to the patient data, we generally select one resistant clone to represent the predominant BCR-ABL1 activity at time zero, that is, at the time of initiation of *dasatinib* or *nilotinib* therapy (which has replaced *imatinib* therapy due to the appearance of resistance).

Following Ref. [23], we differentiate between universal parameters of cancer dynamics, which can be assumed to be identical across patients for a certain CML mutant / drug combination, and patient dependent initial populations. The nature of the universal parameters can further be discriminated between biological, like cell decay rate constants *δ*_*i*_ and *d*_*i*_, and technical, i.e., population thresholds *θ*_*d*_, *θ*_*si*_ as well as clonal competition weights *ω*_*ij*_. Biological and technical parameters can be directly and indirectly linked to individual cellular processes, respectively.

The universal parameters provided in Tables 2 and 3 have been determined in different ways. In Table 2, we follow the similar deterministic models of Refs. [15, 23, 28, 31], and vary relevant starting values in order to qualitatively reproduce certain observed features such as biphasic decline, or delayed appearance followed by rapid growth [40, 41].

**Table 2:** Compilation of coefficients that are common to the test simulations described in Secs. 3.2.1, 3.3 and 3.4.1. The universal biological parameters are defined as follows (*i* = 1, 2, 3): *δ*_*i*_ and *d*_*i*_ are the net death rates of stem cells and differentiated cells, respectively, the prefactors (basic rates of stem cell division) of the stem cell growth functions *ζ*_*i*_(**x**_*s*_(*t*)) are labelled as *α*_*i*_, the symbol for the prefactors of the differentiated cell growth functions *p*(**x**_*d*_(*t*)) (corresponding to differentiation rate constants) is *a*_*i*_, and the rate constants for mutations of the primary resistant stem cell clone *x*_*s*1_(*t*) into the descendant stem cell lines *x*_*s*2_(*t*) and *x*_*s*3_(*t*) are identified by *ν*_2_ and *ν*_3_, respectively. The rate constants for activation of quiescent stem cells and for deactivation of cycling stem cells are denoted by *ν*_*q*1_ and *ν*_*c*1_, respectively. Only indirectly linked to biological properties are the universal technical parameters: the threshold for the differentiated cell growth function *p*(**x**_*d*_(*t*)) is denoted as *θ*_*d*_, the thresholds for the stem cell growth functions *ζ*_*i*_(**x**_*s*_(*t*)) are *θ*_*si*_ and the coefficients that quantify the competition for a stem cell niche between clone *j* (*j* = 1, 2, 3) and other clones *l* are *ω*_*jl*_. Patient dependent parameters are also given: only one set each of initial populations *x*_*sk*_(0) and *x*_*dk*_(0) (*k* = 1, 2, 3) has been employed in all simulations.

Description	Symbol	Value	Variations
Net stem and differentiated cell decay rate constants [per day]	*δ*_*i*_, *d*_*i*_	1.0	*δ*_2_ = *δ*_3_ = 8.0 × 10^−1^
Symmetric division (stem cell birth rate constant) [per day]	*α*_*i*_	1.0	
Asymmetric division (differentiated cell generation rate constant) [per day]	*a*_*i*_	10^6^	
Differentiated cell population threshold [per ml blood]	*θ*_*d*_	10^10^	
Stem cell population threshold	*θ*_*si*_	100.0	*θ*_*s*2_ = 10,100,500
Clonal competition sensitivity weights	*ω*_*ij*_	1.0	*ω*_21_ = *ω*_32_ = 5.0 × 10^−1^
Initial populations, dominant clone [stem cells: arbitrary units / differentiated cells: per ml blood]	*x*_*s*1_(0), *x*_*d*1_(0)	100.0, 7.0 × 10^7^	
Initial populations, secondary clone [stem cells: arbitrary units / differentiated cells: per ml blood]	*x*_*s*2_(0), *x*_*d*2_(0)	5.0, 0.0	
Initial populations, tertiary clone [stem cells: arbitrary units / differentiated cells: per ml blood]	*x*_*s*3_(0), *x*_*d*3_(0)	1.0, 0.0	
Rate constants for mutation per cell division [per day]	*ν*_2_, *ν*_3_	0.0, 0.0	
***Models B or C***			
Rate constant of quiescent cell activation [per day]	*ν*_*q*1_	1.0 × 10^−1^	1.0 × 10^−2^
Rate constant of conversion to quiescence [per day]	*ν*_*c*1_	1.0	1.0 × 10^−1^, 5.0 × 10^−1^
Initial populations, quiescent stem cells [arbitrary units]	*x*_*q*1_(0)	100.0	10.0
Stem cell population threshold to initiate quiescence (model C) [per ml blood]	*θ*_*q*1_	1.5 × 10^7^	

**Table 3:** Collection of the coefficients that were employed in the simulations (cf. Sec. 3.4.2) of patient profiles PP14 (Figs 10 and 11) and PP15 (Figs 12–14). For an explanation of the coefficient symbols see the caption of Table 2. This table provides four complete sets of coefficients for a parameterization of Model C. These sets correspond to the simulations presented in Figs 10–13. The graphs shown in Fig 14 represent an exception since this figure has been prepared on the basis of a reduced model C, i.e., the equation system has been truncated from a three-clone to a two-clone model by eliminating Subeqs 8e and 8f from Eq (8). Fig 14 consequently does not include graphs for functions *x*_*s*3_(*t*) and *x*_*d*3_(*t*). Only coefficients related to *x*_*s*1_(*t*), *x*_*s*2_(*t*), *x*_*d*1_(*t*) and *x*_*d*2_(*t*) are therefore defined in the column for Fig 14.

Description	Symbol	Fig 10	Fig 11	Fig 12	Fig 13	Fig 14
Net stem cell decay rate constants [per day]	*δ*_1_	9.5 × 10^−1^	9.5 × 10^−1^	1.0	1.0	9.6 × 10^−1^
	*δ*_2_	1.0	1.0	1.0	1.0	1.0
	*δ*_3_	1.0	1.0	1.0	1.0	-
Stem cell symmetric division rate constants [per day]	*α*_1_	1.0	1.0	1.0	1.0	1.0
	*α*_2_	1.05	1.05	1.018	1.018	1.01
	*α*_3_	1.05	1.05	1.06	1.06	-
Stem cell population thresholds	*θ*_*s*1_	2.0 × 10^−7^	2.0 × 10^−7^	3.03 × 10^−8^	3.0 × 10^−8^	1.0 × 10^−8^
	*θ*_*s*2_	2.0 × 10^−6^	2.0 × 10^−6^	9.68 × 10^−7^	1.0 × 10^−6^	1.6 × 10^−6^
	*θ*_*s*3_	4.0 × 10^−7^	4.0 × 10^−7^	3.33 × 10^−7^	3.5 × 10^−7^	-
Clonal competition sensitivity weights	*ω*_11_	1.0	1.0	1.0	1.0	1.0
	*ω*_12_	1.1 × 10^−1^	1.1 × 10^−1^	-3.0 × 10^−2^	-3.0 × 10^−2^	2.0 × 10^−1^
	*ω*_13_	6.56 × 10^−1^	6.6 × 10^−1^	1.12 × 10^−1^	1.0 × 10^−1^	-
	*ω*_21_	-1.0	-1.0	0.0	0.0	-2.5 × 10^−2^
	*ω*_22_	1.0	1.0	1.0	1.0	1.0
	*ω*_23_	6.3	6.3	2.097	2.0	-
	*ω*_31_	0.0	0.0	0.0	0.0	-
	*ω*_32_	0.0	0.0	0.0	0.0	-
	*ω*_33_	1.0	1.0	1.0	1.0	-
Mutation rate constants [per day]	*ν*_2_	0.0	0.0	0.0	0.0	0.0
	*ν*_3_	0.0	2.0 × 10^−9^	0.0	3.0 × 10^−10^	0.0
Stem cell population threshold to initiate quiescence [BCR-ABL1 / GUS]	*θ*_*q*1_	5.0	5.0	N/A	N/A	0.51
Rate constant of quiescent cell activation [per day]	*ν*_*q*1_	8.0 × 10^−1^	8.0 × 10^−1^	2.5 × 10^−1^	2.5 × 10^−1^	4.0 × 10^−1^
Rate constant of conversion to quiescence [per day]	*ν*_*c*1_	4.75 × 10^−2^	4.75 × 10^−2^	0.0	0.0	4.8
Net differentiated cell decay rate constants [per day]	*d*_1_	1.0	1.0	1.0	1.0	0.9
	*d*_2_	1.0	1.0	1.0	1.0	1.2
	*d*_3_	1.0	1.0	1.0	1.0	-
Differentiated cell generation rate constants [per day]	*a*_1_	2.0 × 10^9^	2.0 × 10^9^	1.0 × 10^8^	1.0 × 10^8^	1.2 × 10^8^
	*a*_2_	1.0 × 10^8^	1.0 × 10^8^	1.0 × 10^8^	1.0 × 10^8^	1.0 × 10^8^
	*a*_3_	1.0 × 10^8^	1.0 × 10^8^	1.0 × 10^8^	1.0 × 10^8^	-
Differentiated cell population thresholds [BCR-ABL1 / GUS]	*θ*_*d*1_	1.0 × 10^1^	1.0 × 10^1^	1.0 × 10^3^	1.0 × 10^3^	1.0 × 10^3^
	*θ*_*d*2_	1.0 × 10^2^	1.0 × 10^2^	1.0 × 10^2^	1.0 × 10^2^	1.0 × 10^2^
	*θ*_*d*3_	1.0 × 10^2^	1.0 × 10^2^	1.0 × 10^2^	1.0 × 10^2^	-
Stem cells: initial populations [arbitrary units]	*x*_*s*1_(0)	4.95 × 10^−7^	4.95 × 10^−7^	1.73 × 10^−7^	1.73 × 10^−7^	1.73 × 10^−7^
	*x*_*s*2_(0)	8.0 × 10^−11^	8.0 × 10^−11^	4.50 × 10^−10^	4.50 × 10^−10^	2.0 × 10^−9^
	*x*_*s*3_(0)	3.0 × 10^−15^	0.0	1.0 × 10^−16^	0.0	-
Differentiated cells: initial populations [BCR-ABL1 / GUS]	*x*_*d*1_(0)	4.95 × 10^1^	4.95 × 10^1^	1.73 × 10^1^	1.73 × 10^1^	1.73 × 10^1^
	*x*_*d*2_(0)	8.0 × 10^−3^	8.0 × 10^−3^	4.5 × 10^−2^	4.5 × 10^−2^	4.5 × 10^−2^
	*x*_*d*3_(0)	0.0	0.0	0.0	0.0	-
Quiescent stem cells: initial population [arbitrary units]	*x*_*q*1_(0)	4.95 × 10^−7^	4.95 × 10^−7^	1.73 × 10^−7^	1.73 × 10^−7^	1.0 × 10^−5^

To reproduce the data provided by PP14 and PP15 of Ref. [41] and to obtain the parameters collected in Table 3, we applied a systematic multi-parameter least-squares fitting procedure to the qualitative simulation parameters as initial values and fit the model functions to the differentiated cell CML clone population data of both patients individually. The also available clinical information on the total leukemic burden at the grid points has not been considered in the fitting process.

The effective cell decay rate constants *δ*_*i*_ and *d*_*i*_ derive from a combination of drug treatment and environmental stresses as well as apoptosis. The *ω*_*ij*_ matrix elements encode clone specific autoregulation via the diagonal *ω*_11_, *ω*_22_ and *ω*_33_ entries, while the off-diagonal *ω*_*ij*_ parameters represent clonal competition.

The asymmetrical choice of *ω*_21_ = *ω*_32_ = 0.5 in the test simulations (cf. Table 2) effectively weakens the competitive effect of clone 2 on clone 1, and of clone 3 on clone 2. This is chosen in order to ensure the possibility of an asymptotic existence of all three stem cell clones, of exclusively *x*_*s*2_(*t*) or *x*_*s*3_(*t*) and of both *x*_*s*2_(*t*) and *x*_*s*3_(*t*). The asymptotic dominance of only *x*_*s*3_(*t*) follows a preceding phase of growth and extinction of *x*_*s*2_(*t*). Disappearance of *x*_*s*2_(*t*) is, however, not induced by competition with *x*_*s*3_(*t*) (cf. Sec. 3.2.1).

### (2.3) Model A: Population dynamics considering three stem cell clone variants

This implementation allows for three resistant mutant subtypes, as would be needed for example for PP7 with Y253H, T315I and F317L as dominant clone subtypes. Following the description of the models in general (see above), functions (*x*_*s*1_(*t*), *x*_*d*1_(*t*), *x*_*s*2_(*t*), *x*_*d*2_(*t*), *x*_*s*3_(*t*), *x*_*d*3_(*t*)) refer to the respective populations of stem and differentiated cells. The stem cells *x*_*si*_ differentiate to produce the blood cells *x*_*di*_. The weights quantifying clone-clone specific competition between the different stem cell clones are given by the off-diagonal elements of the 3x3 array ***ω*** ≔ {*ω*_*ij*_}. Here, supplementary to the basic model (Eqs (1) and (2)), we allow for one clone (the initially dominant resistant clone) to mutate (“quasi-symmetric” division) to the second and third clone. In this study, nonzero mutation rates will be taken into account only in the patient simulations (cf. Table 3), not in the test simulations (cf. Table 2).

The following coupled system of six first order nonlinear differential equations represents the dynamics of the six cell populations and defines model A:

$$\begin{array}{ccc} \frac{\text{d}x_{s1}\left(t\right)}{\text{d}t} & = & (1-\nu_{2}-\nu_{3})\zeta_{1}\left(x_{s}\left(t\right)\right)\alpha_{1}x_{s1}\left(t\right)-\delta_{1}x_{s1}\left(t\right)\;, \end{array}$$ (4a)

$$\begin{array}{ccc} \frac{\text{d}x_{d1}\left(t\right)}{\text{d}t} & = & p\left(x_{d}\left(t\right)\right)a_{1}x_{s1}\left(t\right)-d_{1}x_{d1}\left(t\right)\;, \end{array}$$ (4b)

$$\begin{array}{ccc} \frac{\text{d}x_{s2}\left(t\right)}{\text{d}t} & = & \nu_{2}\alpha_{1}\zeta_{1}\left(x_{s}\left(t\right)\right)x_{s1}\left(t\right)+\zeta_{2}\left(x_{s}\left(t\right)\right)\alpha_{2}x_{s2}\left(t\right)-\delta_{2}x_{s2}\left(t\right)\;, \end{array}$$ (4c)

$$\begin{array}{ccc} \frac{\text{d}x_{d2}\left(t\right)}{\text{d}t} & = & p\left(x_{d}\left(t\right)\right)a_{2}x_{s2}\left(t\right)-d_{2}x_{d2}\left(t\right)\;, \end{array}$$ (4d)

$$\begin{array}{ccc} \frac{\text{d}x_{s3}\left(t\right)}{\text{d}t} & = & \nu_{3}\alpha_{1}\zeta_{1}\left(x_{s}\left(t\right)\right)x_{s1}\left(t\right)+\zeta_{3}\left(x_{s}\left(t\right)\right)\alpha_{3}x_{s3}\left(t\right)-\delta_{3}x_{s3}\left(t\right)\;, \end{array}$$ (4e)

$$\begin{array}{ccc} \frac{\text{d}x_{d3}\left(t\right)}{\text{d}t} & = & p\left(x_{d}\left(t\right)\right)a_{3}x_{s3}\left(t\right)-d_{3}x_{d3}\left(t\right)\;, \end{array}$$ (4f)

where the parameters *δ*_*i*_ and *d*_*i*_ are the net decay rate constants of stem and differentiated cells, respectively; *α*_*i*_ and *a*_*i*_ are the symmetric and asymmetric division rate constants, modulated by the population dependent growth or negative feedback switch functions *ζ*_*i*_(**x**_*s*_(*t*)) and *p*(**x**_*d*_(*t*)), respectively; and the rate constants for mutation of clone 1 to clones 2 and 3 during “quasi-symmetric” division are *ν*_2_ and *ν*_3_, respectively. The negative feedback function for the generation of differentiated cells switches off asymmetric division exponentially as the total *x*_*di*_ population exceeds threshold *θ*_*d*_:

$$\begin{array}{c} p\left(x_{d}\left(t\right)\right)≔\exp(-\frac{\sum_{i=1}^{3}x_{di}\left(t\right)}{\theta_{d}})\;, \end{array}$$ (5)

The corresponding functions for stem cell symmetric division are:

$$\begin{array}{ccc} \zeta_{1}\left(x_{s}\left(t\right)\right) & ≔ & \exp(-\frac{\omega_{11}x_{s1}\left(t\right)+\omega_{12}x_{s2}\left(t\right)+\omega_{13}x_{s3}\left(t\right)}{\theta_{s1}})\;, \end{array}$$ (6a)

$$\begin{array}{ccc} \zeta_{2}\left(x_{s}\left(t\right)\right) & ≔ & \exp(-\frac{\omega_{21}x_{s1}\left(t\right)+\omega_{22}x_{s2}\left(t\right)+\omega_{23}x_{s3}\left(t\right)}{\theta_{s2}})\;, \end{array}$$ (6b)

$$\begin{array}{ccc} \zeta_{3}\left(x_{s}\left(t\right)\right) & ≔ & \exp(-\frac{\omega_{31}x_{s1}\left(t\right)+\omega_{32}x_{s2}\left(t\right)+\omega_{33}x_{s3}\left(t\right)}{\theta_{s3}})\;. \end{array}$$ (6c)

Here, each clone may respond differently to the populations of each of the subclone types, reflecting the likelihood that clonal competition involves signalling that differs between the ABL1 variants, and symmetric division is switched off as the weighted sum of *x*_*si*_ populations exceeds the threshold *θ*_*si*_. The effect on the proliferation of stem cells is inhibitory if the *ω*_*ij*_ constants take positive values, and is enhancing for negative values.

**Fig 1:**
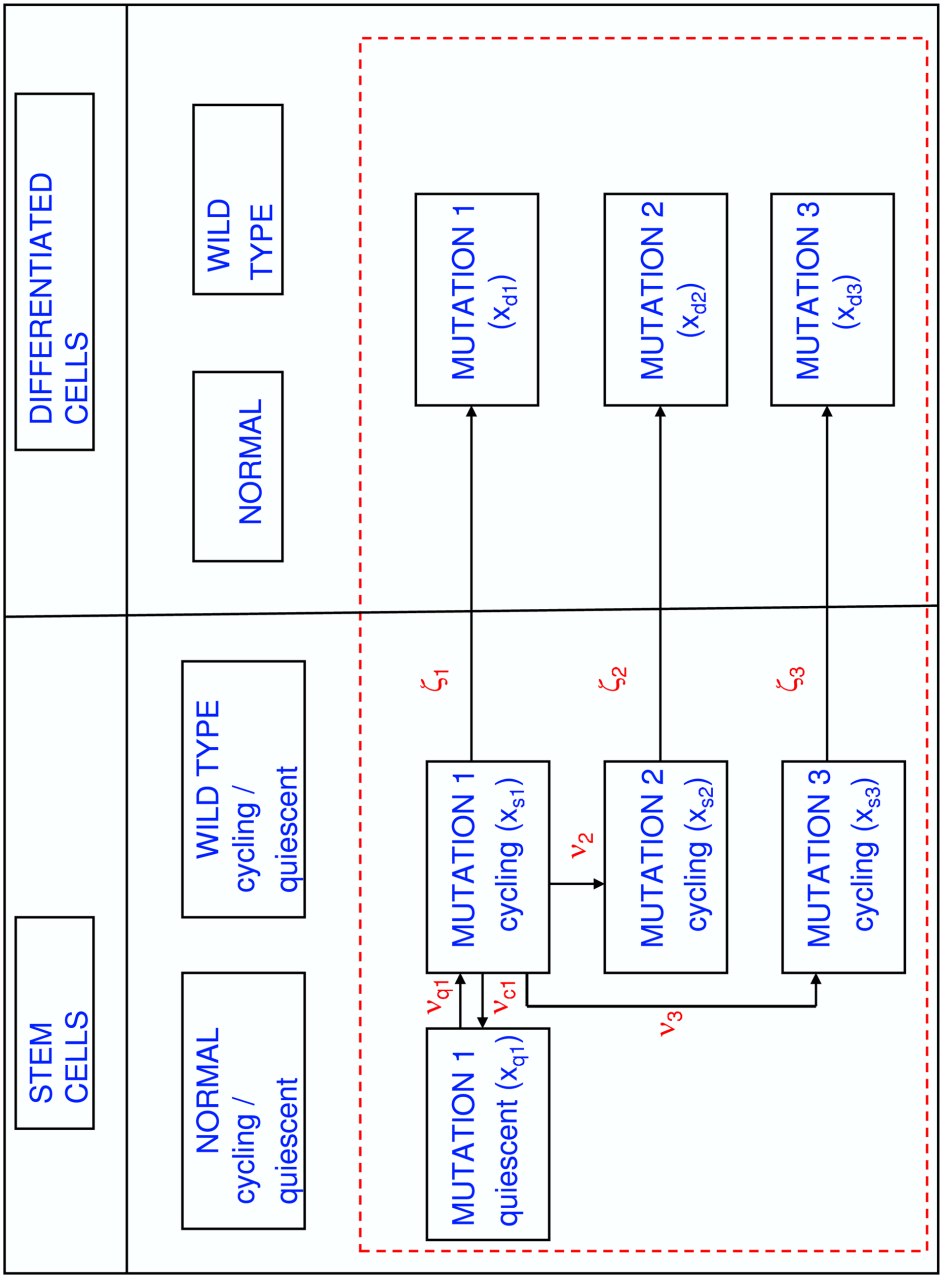
Schematic overview of key characteristics of the computational models developed in Secs. 2.3, 2.4, and 2.5. All models feature a stem and a differentiated cell compartment. The differentiated cell compartment integrates the three layers of progenitor, differentiated and terminally differentiated cells. In both compartments, normal and wild-type CML cell populations are treated as stable in time over the simulation period, which corresponds to administration of *dasatinib* or *nilotinib*, and are not included in the dynamical models. Competition for resources is restricted to three *imatinib*-resistant clones of cycling stem cells (*x*_*s*1_(*t*), *x*_*s*2_(*t*), *x*_*s*3_(*t*)) and differentiated cells (*x*_*d*1_(*t*), *x*_*d*2_(*t*), *x*_*d*3_(*t*)). The rates of symmetric and asymmetric stem cell division are controlled by the growth functions *ζ*_*i*_(**x**_*s*_(*t*)) defined in Eq (6). Only mutations of the initially dominant clone *x*_*s*1_(*t*) into *x*_*s*2_(*t*) and *x*_*s*3_(*t*) are taken into account, the relevant rate constants are *ν*_2_ and *ν*_3_, respectively. The individual models are distinguished by the way in which stem cells of type mutation 1 are interconverting between the growth environments “quiescent” (*x*_*q*1_(*t*)) and “cycling” (*x*_*s*1_(*t*)), the characteristic rate constants are *ν*_*q*1_ and *ν*_*c*1_. Quiescent stem cells reservoirs for mutations 2 and 3 are not included in the model.

A graphical representation of model A can be found as a special case (no quiescence) of Fig 1. Although this model includes terms for mutation of one clone type into another, clinical and experimental data indicate that rate constants for mutations per cell division are very small. The mutation rate constants obtained from the patient simulations performed for this study (cf. Table 3) are in a similar range as the ones reported in Ref. [23].

Mutation rate constants may be small but the patient profiles presented in Refs. [38, 40, 41] demonstrate the relevance of transformations from the wild-type cancer into the *imatinib*-resistant stem cell clones *x*_*s*1_(*t*), *x*_*s*2_(*t*) and *x*_*s*3_(*t*) that occured during or prior to *imatinib* treatment. The preceding resistance mutations are the basis for the choice of nonzero initial populations *x*_*s*1_(0), *x*_*d*1_(0), *x*_*s*2_(0) and *x*_*s*3_(0) in all test simulations (cf. Table 2). Similarly, *x*_*s*1_(0), *x*_*d*1_(0), *x*_*s*2_(0) and *x*_*d*2_(0) are generally assumed to be nonzero in the patient simulations, however, nonzero values of *x*_*s*3_(0) are adopted only in two out of the four calculations involving all three resistant clones. The two cases with *x*_*s*3_(0) = 0 correspond to situations with nonzero mutation rate constants *ν*_3_ (cf. Table 3).

### (2.4) Model B: Modification of Model A to include a quiescent stem cell pool with spontaneous transformation into the cycling state

A simple ansatz to include the effect of a quiescent stem cell population *x*_*q*1_(*t*) is to assume a contribution to the *x*_*s*1_(*t*) population with a proportional rate constant *ν*_*q*1_. This assumes that the quiescent stem cell population is spontaneous, insensitive to any signals for a transformation to a cycling state, and results in the depletion of the quiescent pool, so the flexibility of this model is limited. This extended model for the CML population dynamics becomes a system of seven coupled nonlinear differential equations; five unmodified from the general model A described by system Eq (4), a modification of the equation for $\frac{\text{d}x_{s1}\left(t\right)}{\text{d}t}$ (Eq (4a)), and an additional equation for $\frac{\text{d}x_{q1}\left(t\right)}{\text{d}t}$:

$$\begin{array}{ccc} \frac{\text{d}x_{s1}\left(t\right)}{\text{d}t} & = & (1-\nu_{2}-\nu_{3})\zeta_{1}\left(x_{s}\left(t\right)\right)\alpha_{1}x_{s1}\left(t\right)-\delta_{1}x_{s1}\left(t\right)+\nu_{q1}x_{q1}\left(t\right)\;,\\ \vdots & & \end{array}$$ (7a)

$$\begin{array}{ccc} \frac{\text{d}x_{q1}\left(t\right)}{\text{d}t} & = & -\nu_{q1}x_{q1}\left(t\right)\;. \end{array}$$ (7g)

This model is represented as a special case in Fig 1 whereby cycling cells do not become quiescent.

### (2.5) Model C: Modification of Model B to transform cycling stem cells into quiescence as a function of the total differentiated cell population

Here, we suggest that there is a feedback signal to shift stem cells into a quiescent state if the total population of differentiated cells is larger than a target threshold value, and modify model B to allow for bidirectional exchange between the *x*_*s*1_(*t*) and *x*_*q*1_(*t*) cell pools. To do this, Eqs (7a) and (7g) of model B are replaced by Eqs (8a) and (8g), respectively, using a Heaviside step function to trigger partial conversion to quiescence when $\sum_{j=1}^{3}x_{dj}\left(t\right)>\theta_{q1}$:

$$\begin{array}{ccc} \frac{\text{d}x_{s1}\left(t\right)}{\text{d}t} & = & (1-\nu_{1}-\nu_{2})\zeta_{1}\left(x_{s}\left(t\right)\right)x_{s1}\left(t\right)-[\delta_{1}+\nu_{c1}H(\frac{\sum_{j=1}^{3}x_{dj}\left(t\right)}{\theta_{q1}}-1)]x_{s1}\left(t\right)+\nu_{q1}x_{q1}\left(t\right)\;,\\ \vdots & & \end{array}$$ (8a)

$$\begin{array}{ccc} \frac{\text{d}x_{q1}\left(t\right)}{\text{d}t} & = & \nu_{c1}H(\frac{\sum_{j=1}^{3}x_{dj}\left(t\right)}{\theta_{q1}}-1)x_{s1}\left(t\right)-\nu_{q1}x_{q1}\left(t\right)\;. \end{array}$$ (8g)

Model C represents the complete scheme shown in Fig 1. The system Eq (8) has been employed in the test simulations described in Sec. 3.4. However, numerical difficulties linked to the Heaviside function have been encountered when employing Eq (8) for the patient simulations outlined in Sec. 3.4. In the patient simulations, the Heaviside function *H*(*x*) appearing in Eqs (8a) and (8g) is therefore replaced by the sigmoidal function *σ*(*x*) defined as:

$$\begin{array}{c} \sigma \left(x\right)=\frac{1}{1+\exp(-c_{1}\left(x-c_{2}\right))}\;, \end{array}$$ (9)

where *c*_1_ and *c*_2_ are parameters. With the definitions *c*_1_ ≔ 100 and *c*_2_ ≔ 0, the function *σ*(*x*) represents a close approximation to *H*(*x*).

## Results

### (3.1) Details of the simulations

With the three models described above, we vary key parameters (cf. Tables 2 and 3) and examine their effects on clone dynamics, compare with clinical data, and consider potential conclusions regarding stem cell properties. In the general simulations, the models are parameterized such that the differentiated cell decay rate constants *d*_*i*_ are identical (1.0) for all three clones, but the stem cell decay rate constants are nonuniform: the relevant parameter *δ*_1_ for the initially (*imatinib*-resistant) dominant clone (1.0) is higher than that for the clones (*δ*_2_ = *δ*_3_ = 0.8) that come to replace it under new therapy (treatment with *dasatinib* or *nilotinib*). The initial cycling stem cell populations *x*_*si*_(0) assume a large value for the dominant clone (100.0) compared to the secondary (5.0) or tertiary (1.0) clones, values that reflect differential sensitivities to *imatinib*. Further, clonal competition is parameterized such that the secondary clone is relatively insensitive to the dominant clone, and similarly, the tertiary clone is relatively insensitive to the secondary clone. This is not symmetric: the primary clone is fully sensitive to competition with the secondary clone, as is the secondary clone to the tertiary clone.

The parameter variations test differential sensitivities to clonal competition, initial populations, and exchange rates between quiescent and cycling stem cell populations (models B and C). Mutation rates are also examined in the patient simulations with model C. Key patterns of clone dynamics in patient data to be reproduced include especially the rapid but sometimes bi- or multiphasic clearance of the initial *imatinib*-resistant clone, and the sequential appearance of two apparently competing *dasatinib*- or *nilotinib*-resistant clones (cf. Table 1).

Rapid clearance of the initial clone may be achieved with a lower decay rate for the secondary and tertiary resistant differentiated cell clones, while a quiescent stem cell pool provides a mechanism to explain biphasic or more complex clearance patterns seen in patients. Differential sensitivity to clonal competition, coupled with initial population values, can explain the pattern of sequential appearance of new resistant clones.

All models are tested for sensitivity to clonal competition by varying *θ*_*s*2_ (sensitivity of clone 2 to the total stem cell population) from 10% to 500% of the *θ*_*si*_ values for the other two clones. The three models differ with respect to effects of a quiescent stem cell state of clone 1: Model A has none, model B has a quiescent pool with a first order irreversible depletion rate, and model C allows for an equilibrium between quiescent and cycling states, controlled by feedback signalling of the total differentiated cell population.

Little information is available on the ratio between cycling and non-cycling growth environments of hematopoietic stem cells. This ratio seems likely to differ between normal and variants of leukemic stem cells. In the computational study Ref. [28], the effect of the addition of Granulocyte-Colony Stimulating Factor (G-CSF) to *imatinib* has been simulated, assuming different cycling-to-non-cycling ratios of wild-type CML stem cells. A comparison of the results with observations of the clinical study Ref. [50], which is based on a small patient cohort, suggests that the majority of wild-type CML stem cells is cycling.

In the test simulations, the ratio *x*_*s*1_(0) / *x*_*q*1_(0) between the starting populations of active and quiescent stem cells of the initially dominant clone has been defined as either 1.0 or 10.0 (cf. Table 2). For the five patient simulations discussed in Sec. 3.4.2, values of 1.0 or 0.0173 have been adopted for the ratio *x*_*s*1_(0) / *x*_*q*1_(0) (cf. Table 3).

**Fig 2:**
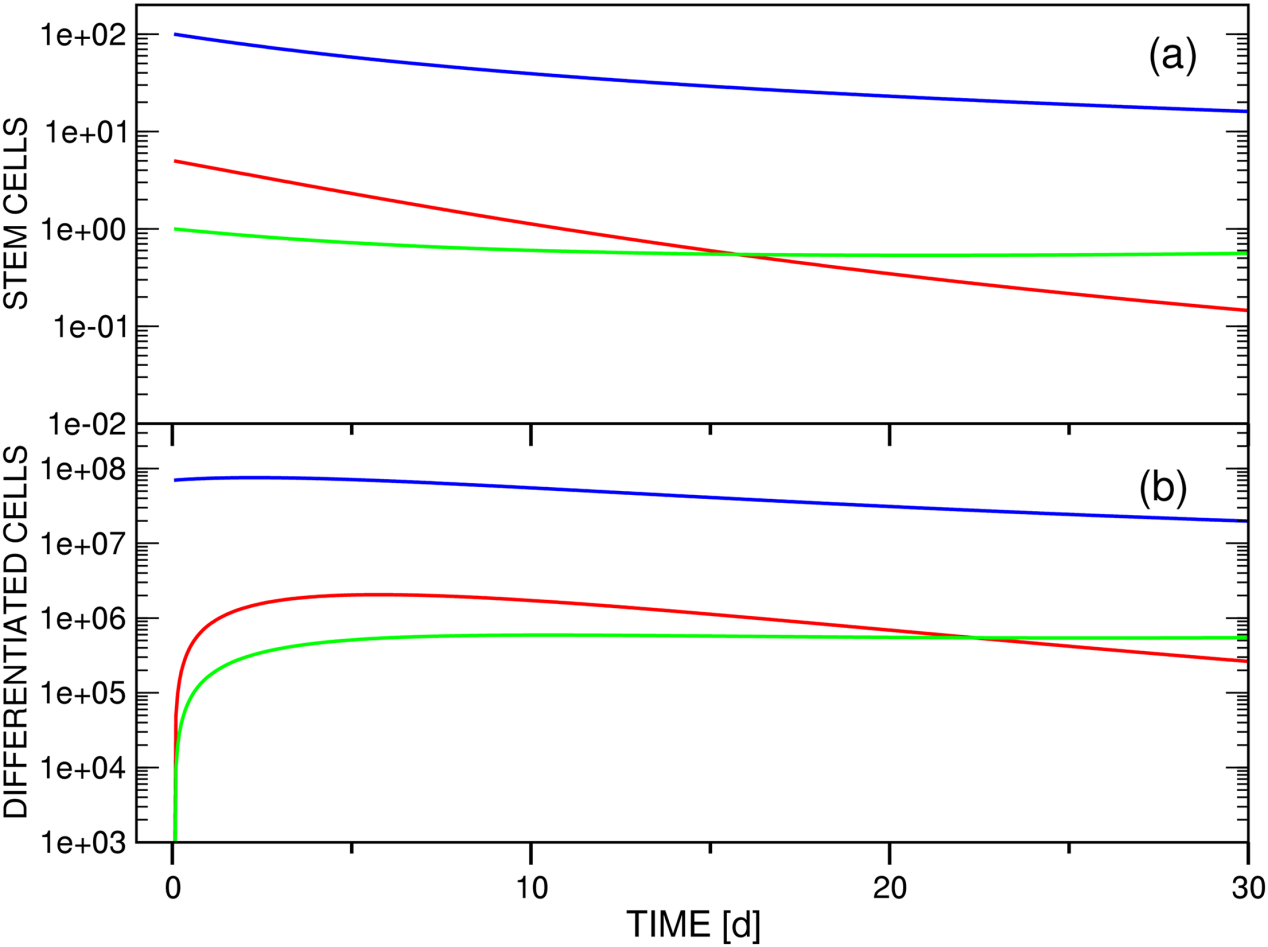
Test simulation of the competition dynamics of three *imatinib*-resistant stem cell clones (a) and of the associated differentiated cell populations (b) employing model A (Eq (4)). The focus is on the initial phase of *dasatinib* treatment, in which an equilibrium between the populations of stem and differentiated cells is established. In panel (b), the time axis corresponds to the interval [0d,30d] of panel Fig 3(a). Accordingly, the threshold setting is *θ*_*s*2_ = 10.0. The other parameters employed for the simulation are compiled in Table 2. Color scheme: *x*_*s*1_ / *x*_*d*1_ (blue), *x*_*s*2_ / *x*_*d*2_ (red), *x*_*s*3_ / *x*_*d*3_ (green). The ordinate units of panels (a) and (b) are arbitrary and number of cells / ml blood, respectively.

Excepting short phases of rapid variation during responses to initial conditions at very early times (cf. Fig 2), the modelled differentiated cell populations are in a state of quasi-static equilibrium with the cancer stem cells, due to the relatively slow time scale of stem cell proliferation. This view holds when equilibration occurs rapidly compared to the other rates, and for our model, we obtain the quasi-static relationships between the stem and differentiated cell populations by approximating the time derivatives as zero, giving:

$$\begin{array}{c} x_{di}\left(t\right)\approx \exp(-\frac{\sum_{j=1}^{3}x_{dj}\left(t\right)}{\theta_{d}})\frac{a_{i}}{d_{i}}x_{si}\left(t\right)\;, \end{array}$$ (10)

where, as described in Sec. 2.1, the exponential factor represents the switching function that downregulates differentiation as a function of the total population of differentiated cells, *a*_*i*_ is the constant determining the rate at which stem cell differentiation via asymmetric division occurs [31], and *d*_*i*_ is the decay rate constant for the differentiated cell population *x*_*di*_(*t*). This relationship shows the proportionality that approximates the relative populations of stem and differentiated cells at total differentiated cell populations far below the threshold for switching off asymmetric division, and how this proportionality is reduced when the total populations surpass the threshold. Thus, in the analysis of results below, the behavior of the stem cell populations may be inferred from the behavior of the differentiated cells very soon after the time origin. This assumption of a direct proportionality between the populations of stem and differentiated cells of individual CML clones is corroborated by clinical observations [17].

### (3.2) Model A: Three mutated stem cell clones with no quiescence

#### (3.2.1) Mechanistic interpretation (Figs 2 and 3)

**Fig 3:**
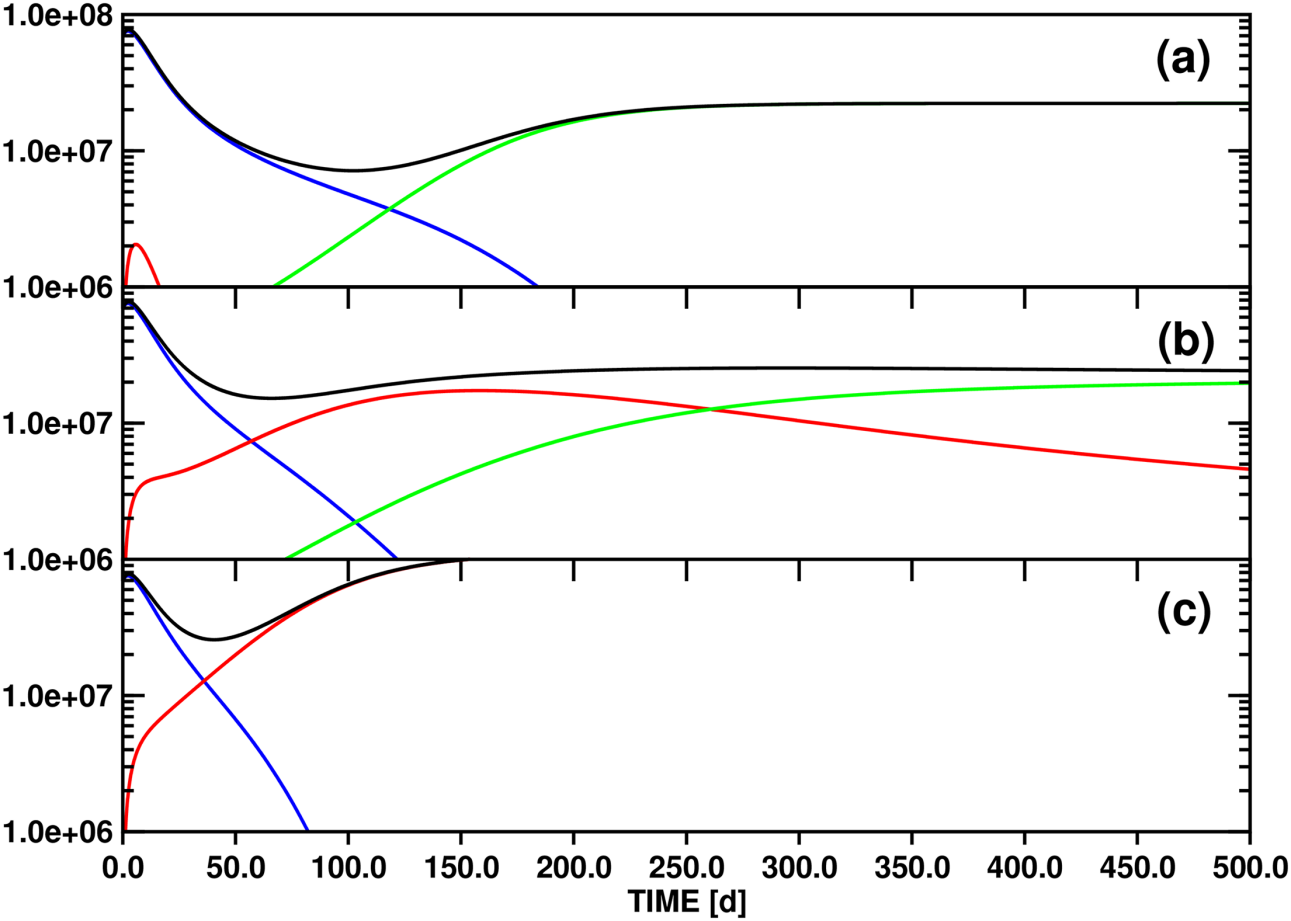
Test simulations of the population dynamics of three *imatinib*-resistant differentiated cell clones and of the total number of differentiated cells based on model A (Eq (4)). The panels (a), (b) and (c) correspond to the threshold settings *θ*_*s*2_ = 10.0, 100.0 and 500.0, respectively. The other parameters employed for the simulations are compiled in Table 2. Color scheme: *x*_*d*1_ (blue), *x*_*d*2_ (red), *x*_*d*3_ (green) and *y* (black). The ordinate unit is number of cells / ml blood.

Parameter variation for model A generates scenarios for the replacement of the initially dominant clone by either secondary or tertiary clones, depending in part on sensitivity to clonal competition. Fig 3 shows the evolution of the individual differentiated cell lines *x*_*d*1_(*t*), *x*_*d*2_(*t*), *x*_*d*3_(*t*), and of the total population *y*(*t*), over a 500d time interval for three values (10.0, 100.0, 500.0) of the threshold stem cell population *θ*_*s*2_ that slows asymmetric division of clone 2 (compared to 100.0 for *θ*_*s*1_ and *θ*_*s*3_). The other model parameters are given in Table 2.

Because of an imbalance between the starting populations of stem and differentiated cells imposed by the definitions compiled in Table 2, all test simulations are characterized by a short initial equilibration phase. For model A (Fig 3), the population of the initially dominant strain *x*_*d*1_(*t*) increases by ca. 8% to reach quasi-static equilibrium and a maximum after 2d. As a result of the choice of nonzero and zero initial populations of stem (*x*_*s*2_(0), *x*_*s*3_(0)) and differentiated cells (*x*_*d*2_(0), *x*_*d*3_(0)), respectively, the two differentiated cell functions jump from zero to ∼1.0 × 10^5^ (*x*_*d*2_(*t*)) and ∼1.0 × 10^4^ (*x*_*d*3_(*t*)) initially, and stabilize to levels consistent with Eq (10). Fig 2 shows this for the case *θ*_*s*2_ ≔ 10.0 on the interval [0d,30d].

Following equilibration, an initially rapid exponential decline of *x*_*d*1_(*t*) proceeds for 30d-50d, followed by a regime of slower decay as other strains acquire significant populations. This biphasic decline pattern is independent of the value of *θ*_*s*2_ (Fig 3).

For *θ*_*s*2_ ≔ 10.0 (Fig 3a), the test value with the greatest sensitivity of clone 2 to the total differentiated cell population *y*(*t*), the small differentiated cell population *x*_*d*2_(*t*) appears and disappears quickly, as equilibration is followed by the inhibitory effects of the population *x*_*s*1_(*t*) on *x*_*s*2_(*t*) alone, via *ζ*_2_(**x**_*s*_(*t*)). The rate of exponential decline of *x*_*d*1_(*t*) slows as the total stem cell population drops, diminishing the feedback signal to block symmetric division. After 120d, *x*_*d*3_(*t*) surpasses *x*_*d*1_(*t*), reaching a stable value as the only surviving differentiated cell type after ca. 250d. The later appearance of *x*_*d*3_(*t*) compared to *x*_*d*2_(*t*) is an effect of the smaller initial population *x*_*s*3_(0) of the parent stem cell strain. In contrast to clone 2, which is highly sensitive to clonal competition, clones 1 and 3 share equal sensitivity to each other. Thus, in the absence of a significant *x*_*s*2_ population, clone 3 outcompetes clone 1 due to its greater resistance to the new drug regime (as modelled by the lower decay rate, *δ*_1_ > *δ*_3_).

With *θ*_*s*2_ ≔ 100.0 (Fig 3b), variations in the *x*_*si*_ sensitivity to total stem cell population arise solely from the clone-clone specific interaction sensitivities parameterized by *w*_*ij*_. In this case, both clones 2 and 3 survive into the long term regime, with at least clone 3 and probably both approaching a stable asymptote (see discussion below). The higher initial population *x*_*s*2_(0) as compared to *x*_*s*3_(0) leads to transient dominance of clone 2, but the relative insensitivity of clone 3 to competition from clone 2 via *ω*_32_ ≔ 0.5 leads to its predominance by about 250d. These trends are much greater for *θ*_*s*2_ ≔ 500.0 (Fig 3c); with low general sensitivity to clonal competition for clone 2, it rapidly achieves dominance, and clone 3 never appears.

#### (3.2.2) Mathematical analysis

Fig 3 demonstrates the interesting phenomenon that three different long-time scenarios are realized in the state space spanned by *x*_*d*1_(*t*), *x*_*d*2_(*t*) and *x*_*d*3_(*t*) by varying *θ*_*s*2_ from 10.0 to 500.0: (i) for *θ*_*s*2_ ≔ 10.0, only the *x*_*d*3_(*t*) population converges to a nonzero limit, (ii) for *θ*_*s*2_ ≔ 100.0, both *x*_*d*2_(*t*) and *x*_*d*3_(*t*) maintain nonzero values after 500d, although it is not obvious whether *x*_*d*2_(*t*) will eventually disappear or not, and (iii) for *θ*_*s*2_ ≔ 500.0, only *x*_*d*2_(*t*) survives asymptotically. This observation indicates that variation of *θ*_*s*2_ may determine the state of the system with respect to (at least) two bifurcation points, i.e., the locations in parameter space at which the asymptotic behaviors change.

The global dynamics of systems similar to Eqs (4), (7) and (8) have been investigated in Refs. [31, 33, 34]. Wodarz [31] analyzed a basic model for populations of healthy and CML stem and differentiated cells, including a term for uncontrolled stem cell division. This 4D equation system is most similar to model A since (i) both stem and differentiated cell compartments are included and (ii) exponential growth functions are employed for both stem and differentiated cell clones featuring negative feedback induced by total population of the respective cell type. Wodarz’ basic model also allows for variation of symmetric interclonal competition weights (through the “*ϵ*” parameter) but differs structurally from Eq (4) in that a term representing blast phase development is added to the subequation describing time evolution of the CML stem cell clone. The blast phase term is a function of the total population of differentiated cells and therefore couples the differentiated and stem cell compartments. The dynamics of differentiated cells depends of course on the stem cell populations in models A, B, C and in all models of Ref. [31].

The systems of Refs. [33, 34] consider only the stem cell compartments, but are analogous to models A and B of this work, because the dynamics governed by Eqs (4) and (7) is independent of differentiated cell populations. This analogy does not apply to model C, because the terms determining activation and deactivation of stem cell clone 1 (functions *x*_*s*1_(*t*), *x*_*q*1_(*t*)) in Eqs (8a) and (8g) depend on the total population of differentiated cells.

Each of the three models defined in Refs. [33, 34] also differ in the choice of stem cell growth functions, replacing the exponential growth functions *ζ*_*i*_(**x**_*s*_(*t*)) by sigmoidal functions either (i) of Hill type (Ref. [33]) or (ii) formulated in terms of Tsallis q-exponential expressions (Ref. [34]).

In Ref. [31], the steady state properties of the system of two competing stem cell clones without a feedback signal from differentiated cell populations (blast phase term) have been investigated (this 2D equation system is referred to as Wodarz’ reduced model in the following). Two scenarios occur depending on variation of “*ϵ*”: (i) If the intra- and interclonal competition weights for both strains are equal, then only the clone with the largest “birth rate” constant *η* survives. (ii) However, if *ϵ* is adjusted such that intraclonal competition is greater than interclonal competition, then three long term solutions are possible: survival of either clone alone, or coexistence of both stem cell populations. Accordingly, two bifurcation points can be identified in that parameter space.

Scenario (ii) is reminiscent of the pattern represented by Fig 3, assuming proportionality between stem and differentiated cell populations and disregarding the rapidly vanishing *x*_*d*1_(*t*) / *x*_*s*1_(*t*) populations. In fact, with the respective parameter definitions, the contest of three stem cell clones formulated by model A is effectively scaled down on longer time scales to a 2D problem involving *x*_*s*2_(*t*) and / or *x*_*s*3_(*t*) due to the conclusive elimination of *x*_*s*1_(*t*) beyond 200d (linked to the disappearance of *x*_*d*1_(*t*), cf. Fig 3), independent of the value of *θ*_*s*2_. Starting from the 3D stem cell system represented by Eqs (4a), (4c) and (4e) and defining *x*_*s*1_(*t*) = 0.0, *x*_*d*1_(*t*) = 0.0, *ν*_2_ ≔ 0.0 and *ν*_3_ ≔ 0.0, we obtain the 2D equation system:

$$\begin{array}{ccc} \frac{\text{d}x_{s2}\left(t\right)}{\text{d}t} & = & (\exp(-\frac{\omega_{22}x_{s2}\left(t\right)+\omega_{23}x_{s3}\left(t\right)}{\theta_{s2}})\alpha_{2}-\delta_{2})x_{s2}\left(t\right)\;, \end{array}$$ (11a)

$$\begin{array}{ccc} \frac{\text{d}x_{s3}\left(t\right)}{\text{d}t} & = & (\exp(-\frac{\omega_{32}x_{s2}\left(t\right)+\omega_{33}x_{s3}\left(t\right)}{\theta_{s3}})\alpha_{3}-\delta_{3})x_{s3}\left(t\right)\;, \end{array}$$ (11b)

that corresponds to the reduced model of Ref. [31]. A comparison of both 2D equation systems shows that *ϵ* and *β* of Ref. [31] are equivalent to *ω*_*ij*_ and 1/*θ*_*si*_, respectively, if *ω*_*ii*_ ≔ 1.0.

In this context, it is helpful to differentiate two qualities that may both depend on the value of a parameter: (i) determination of the number of equilibria and (ii) connection of different equilibria via bifurcation points.

With the parameter definitions of Ref. [31], *ϵ* combines both qualities. Setting *ϵ* ≔ 1.0 implies the existence of two single-state equilibria, which equilibrium is realized depends on the choice of other parameters (this property of *ϵ* corresponds to option (i)). A value *ϵ*<1.0 entails three mutually exclusive equilibria, and the three equilibria can be connected by tuning *ϵ* while the remaining parameters are kept frozen (this property of *ϵ* corresponds to both options (i) and (ii)).

Inspection of Eq (6) reveals that interclonal competition in the stem cell system described by Eqs (4a), (4c) and (4e) can be maximized only by choosing the *ω*_*ii*_ and *ω*_*ij*_ parameters uniformly for each *ζ*_*i*_(**x**_*s*_(*t*)). *θ*_*s*2_ is thus not a parameter that can determine the number of existing equilibria (corresponding to option (i)). However, under certain conditions (see below) scanning *θ*_*s*2_, with otherwise invariant parameterization, switches between three equilibria (corresponding to option (ii)) which can be stable or unstable.

This result is consistent with the analysis of the reduced 2D stem cell system presented in Ref. [31], i.e., variation of the degree of competition between two stem cell clones via the parameter *ϵ* (Ref. [31]) or *θ*_*s*2_ (this work) produces three equilibria, two of single-state and one of mixed-state character.

A thorough analysis of the global dynamics of 2D stem cell systems analogous to the reduced model of Ref. [31] and to Eq (11), including different choices of growth functions, can be found in Refs. [33, 34]. The dynamics of the 2D models is shown to be completely induced by the equilibria since no admissible values of the parameters for periodic orbits exist [33]. In particular, for both Hill-type [33] and Tsallis-based [34] growth functions, the existence of four isolated equilibria, the origin as well as two of single-state and one of mixed-state type, has been demonstrated, in agreement with the results obtained in Ref. [31] and in the present study. This correspondence reflects the notion that the asymptotic properties of the growth functions determine the nature of the equilibria.

Following Refs. [33, 34], we will analyze the bifurcation scenario of the solutions of Eq (11) in more detail. For convenient comparison we adopt the notation of Eq 2 of Ref. [34] and rewrite Eq (11) in the form:

$$\begin{array}{ccc} \frac{\text{d}\hat{x}\left(t\right)}{\text{d}t} & = & (\exp(-(\hat{x}\left(t\right)+{\hat{\text{w}}}_{1}\hat{y}\left(t\right))){\hat{l}}_{1}-{\hat{d}}_{1})\hat{x}\left(t\right)\;, \end{array}$$ (12a)

$$\begin{array}{ccc} \frac{\text{d}\hat{y}\left(t\right)}{\text{d}t} & = & (\exp(-({\hat{\text{w}}}_{2}\hat{x}\left(t\right)+\hat{y}\left(t\right))){\hat{l}}_{2}-{\hat{d}}_{2})\hat{y}\left(t\right)\;, \end{array}$$ (12b)

with the definitions $\hat{x}\left(t\right)$ = $\frac{\omega_{22}}{\theta_{s2}}x_{s2}\left(t\right)$, $\hat{y}\left(t\right)$ = $\frac{\omega_{33}}{\theta_{s3}}x_{s3}\left(t\right)$, ${\hat{l}}_{1}$ ≔ $\frac{\theta_{s2}}{\omega_{22}}\alpha_{2}$, ${\hat{l}}_{2}$ ≔ $\frac{\theta_{s3}}{\omega_{33}}\alpha_{3}$, ${\hat{d}}_{1}$ ≔ $\frac{\theta_{s2}}{\omega_{22}}\delta_{2}$, ${\hat{d}}_{2}$ ≔ $\frac{\theta_{s3}}{\omega_{33}}\delta_{3}$, ${\hat{w}}_{1}$ ≔ $\frac{\theta_{s3}\omega_{23}}{\theta_{s2}\omega_{33}}$ and ${\hat{w}}_{2}$ ≔ $\frac{\theta_{s2}\omega_{32}}{\theta_{s3}\omega_{22}}$.

The analysis of global dynamics of 2D, 3D and 4D equation systems describing competition between stem cell clones regulated by functions *f*(*u*) presented in Ref. [34] is not restricted to Tsallis-based growth factors but is generally valid for monotonically decreasing functions formulated according to:

$$\begin{array}{c} f\left(u\right)=\frac{1+g_{0}}{1+g(u)}\;. \end{array}$$ (13)

With this convention, the growth functions corresponding to *f*(*u*) employed in Refs. [15, 31] and in the present study are obtained by setting *g*_0_ ≔ 0.0 and *g*(*u*) = exp(*u*)—1.0. Inspection of Eqs (12a) and (12b) reveals that $u=\hat{x}\left(t\right)+{\hat{\text{w}}}_{1}\hat{y}\left(t\right)$ and $u={\hat{\text{w}}}_{2}\hat{x}\left(t\right)+\hat{y}\left(t\right)$, respectively, since *g*(*u*) must be monotonically increasing (i.e., ${\hat{\text{w}}}_{1},{\hat{\text{w}}}_{2}\geq$ 0.0). The discussion of the stability of equilibria of models of stem cell dynamics in Ref. [33] is exclusively considering the assignment *g*(*u*) = *u*^*m*^, i.e., Hill-type growth functions.

In the next step, selection or bifurcation parameters for stem cell clones $\hat{x}\left(t\right)$ and $\hat{y}\left(t\right)$ are specified as *γ*_*x*_ ≔ $\frac{{\hat{l}}_{1}-{\hat{d}}_{1}}{{\hat{d}}_{1}}$ and *γ*_*y*_ ≔ $\frac{{\hat{l}}_{2}-{\hat{d}}_{2}}{{\hat{d}}_{2}}$, respectively. For an analysis of the bifurcation landscape shaped by the solutions of Eq (12) we finally require the characteristic values ${\hat{x}}_{s}≔g^{-1}\left(\gamma_{x}\right)$ and ${\hat{y}}_{s}≔g^{-1}\left(\gamma_{y}\right)$ of the $\hat{x}\left(t\right)$ and $\hat{y}\left(t\right)$ populations (assuming *γ*_*x*_, *γ*_*y*_ > −1.0).

We now have all the necessary tools available to investigate the asymptotic development of the *x*_*d*2_(*t*) and *x*_*d*3_(*t*) population functions as obtained from the three test simulations based on model A (Fig 3). For this purpose we will utilize the property that the functions *x*_*d*2_(*t*) (Eq (4d)) ↔ *x*_*s*2_(*t*) (Eq (4c)) $↔\hat{x}\left(t\right)$ (Eq (12a)) and *x*_*d*3_(*t*) (Eq (4f)) ↔ *x*_*s*3_(*t*) (Eq (4e)) $↔\hat{y}\left(t\right)$ (Eq (12b)) are to a good approximation directly proportional for *t* → ∞.

The relevant numerical values are collected in Table 2. The parameterization of model A yields generally *γ*_*x*_ = *γ*_*y*_ = 0.25 and ${\hat{x}}_{s}$ = ${\hat{y}}_{s}$ = 0.223. Variation of *θ*_*s*2_ then leads to the following scenarios: (i) *θ*_*s*2_ ≔ 10.0 → ${\hat{\text{w}}}_{1}{\hat{y}}_{s}$ = 2.231, ${\hat{\text{w}}}_{2}{\hat{x}}_{s}$ = 0.011 (Fig 3(a)), (ii) *θ*_*s*2_ ≔ 100.0 → ${\hat{\text{w}}}_{1}{\hat{y}}_{s}$ = 0.223, ${\hat{\text{w}}}_{2}{\hat{x}}_{s}$ = 0.111 (Fig 3(b)), and (iii) *θ*_*s*2_ ≔ 500.0 → ${\hat{\text{w}}}_{1}{\hat{y}}_{s}$ = 0.045, ${\hat{\text{w}}}_{2}{\hat{x}}_{s}$ = 0.557 (Fig 3(c)).

Matching the criteria (i), (ii) and (iii) to the conditions labeling each of the 11 panels of Fig 4 of Ref. [34] reveals that parameter configurations (i) and (iii) correspond to the stable line equilibria (0,$\hat{y}\left(t\right)$) ↔ (0, *x*_*d*3_(*t*)) and ($\hat{x}\left(t\right)$, 0) ↔ (*x*_*d*2_(*t*), 0), respectively. While this outcome just confirms the obvious asymptotic limits shown in Fig 3(a) and 3(c), case (ii) is more interesting: the solution of Eq (12) converges again to a line equilibrium (0,$\hat{y}\left(t\right)$) ↔ (0, *x*_*d*3_(*t*)), but in contrast to the stable single-state systems obtained with settings (i) and (iii) the linearization of equilibrium (ii) has a neutral component.

The corresponding panel of Fig 4 of Ref. [34] (at the bottom right) illustrates that convergence trajectories starting from small values ($\hat{x}\left(0\right)$,$\hat{y}\left(0\right)$) ↔ (*x*_*d*2_(0), *x*_*d*3_(0)) as in Fig 3(b) pass through a non-stationary point of inflection ($\hat{x}\left(t_{i}\right)$,$\hat{y}\left(t_{i}\right)$) distinguished by a maximum of the $\hat{x}\left(t\right)$ population. Since this mixed transition state is linked to (*x*_*d*2_(*t*_*i*_), *x*_*d*3_(*t*_*i*_)), we can determine *t*_*i*_ = 158d by locating the maximum of function *x*_*d*2_(*t*) (cf. Fig 3(b)).

According to the classification of equilibria provided in Theorem 1 of Ref. [34], a stable mixed state equilibrium and a line of equilibria cannot be realized for the pair of functions $\hat{x}\left(t\right)↔x_{d2}\left(t\right)$ and $\hat{x}\left(t\right)↔x_{d2}\left(t\right)$ with the parameterization of model A (cf. Table 2) by modulating only *θ*_*s*2_. In fact, stable mixed state equilibria could not be reached even if both *θ*_*s*2_ and *θ*_*s*3_ were allowed to change.

However, variation within the limits 100.0 <*θ*_*s*2_< 200.0 complies with the constraints ${\hat{y}}_{s}>{\hat{\text{w}}}_{2}{\hat{x}}_{s}>0.0\&{\hat{x}}_{s}>{\hat{\text{w}}}_{1}{\hat{y}}_{s}$ and is therefore consistent with unstable mixed state equilibria ($\hat{x}\left(t\right)$,$\hat{y}\left(t\right)$). This analysis proves that the parameterization corresponding to Fig 3(b) (*θ*_*s*2_ ≔ 100.0) represents a bifurcation point separating two asymptotic solutions of Eq (12), the line equilibrium (0,$\hat{y}\left(t\right)$) and the unstable face equilibrium ($\hat{x}\left(t\right)$,$\hat{y}\left(t\right)$). Increasing *θ*_*s*2_ to 200.0 leads to the next bifurcation point dividing the regime of the unstable mixed state ($\hat{x}\left(t\right)$,$\hat{y}\left(t\right)$) from the stable single-state equilibrium ($\hat{x}\left(t\right)$, 0).

Information on the bifurcation landscape is of great value for an understanding of the dynamics governed by an equation system since the sensitivity of the solutions to variations of certain parameters reaches a maximum at such a branch point. With a parameterization of Eq (12) corresponding to Table 2, tuning *θ*_*s*2_ in to the values 100.0 and 200.0 will therefore yield the strongest dependence of the solutions on this parameter.

Of interest in this context is also how the parameterization of Eq (12) imposed by Table 2 needs to be modified in order for the solutions to converge to stable mixed state equilibria ($\hat{x}\left(t\right)$,$\hat{y}\left(t\right)$). To provide a numerical example, the condition ${\hat{\text{w}}}_{2}{\hat{x}}_{s}>{\hat{y}}_{s}>0.0\&{\hat{x}}_{s}<{\hat{\text{w}}}_{1}{\hat{y}}_{s}\;$, in combination with the requirement that the characteristic values ${\hat{x}}_{s}$ and ${\hat{y}}_{s}$ as well as the coefficients *ω*_22_ and *ω*_33_ should be conserved, defines the inequalities *ω*_32_> 4.484 and *ω*_23_> 0.223 for any choice satisfying *θ*_*s*2_ = *θ*_*s*3_.

Inspection of Fig 4 of Ref. [34] shows that the characteristic values (${\hat{x}}_{s}^{0},{\hat{y}}_{s}^{0}$) at a particularly critical bifurcation point are generally defined by the constraint ${\hat{\text{w}}}_{2}{\hat{x}}_{s}^{0}={\hat{y}}_{s}^{0}>0.0\&{\hat{x}}_{s}^{0}={\hat{\text{w}}}_{1}{\hat{y}}_{s}^{0}$. Depending on the orientation of any deviation from this focal point in the multidimensional parameter space, one of multiple scenarios will ensue for the asymptotic solutions of Eq (12).

With the parameter definitions of Eq (11), we obtain the following identity at the focal bifurcation point: (${\hat{x}}_{s}^{0},{\hat{y}}_{s}^{0}$) = $\left(\frac{\theta_{s3}\omega_{23}^{2}\omega_{32}}{\theta_{s2}\omega_{33}^{2}\omega_{22}},\frac{\omega_{32}\omega_{23}}{\omega_{22}\omega_{33}}\right)$, i.e., the complete set of growth function parameters determines ${\hat{x}}_{s}^{0}$ and ${\hat{y}}_{s}^{0}$ or *vice versa*. Inserting, e.g., the coefficients *θ*_*s*2_, *θ*_*s*3_, *ω*_22_, *ω*_33_, *ω*_23_, *ω*_32_ employed for preparation of Fig 3(b) results in the characteristic values (${\hat{x}}_{s}^{0},{\hat{y}}_{s}^{0}$) = (0.5, 0.5). This unstable equilibrium thus corresponds to a mixed state with equal populations of clones $\hat{x}\left(t\right)$ and $\hat{y}\left(t\right)$.

However, full parameterization of Eq (11) requires in addition the rate constants *α*_2_, *α*_3_, *δ*_2_ and *δ*_3_ which determine the characteristic values ${\hat{x}}_{s}$ and ${\hat{y}}_{s}$. If we again refer to the set of coefficients defined for the simulation illustrated in Fig 3(b) then we calculate: ${\hat{x}}_{s}$ = ${\hat{y}}_{s}$ = 0.223.

This comparison confirms that the parameters employed for computation of the functions *x*_*d*2_(*t*) and *x*_*d*3_(*t*) shown in Fig 3(b) are consistent with a bifurcation point separating only two scenarios, the single-state and the unstable mixed-state equilibrium (0,$\hat{y}\left(t\right)$) and ($\hat{x}\left(t\right)$,$\hat{y}\left(t\right)$), respectively. A multivalent focal bifurcation point could for example be reached for the solutions of Eq (11) via modification of the coefficients *α*_2_, *α*_3_, *δ*_2_ and *δ*_3_ to comply with the condition (${\hat{x}}_{s}^{0},{\hat{y}}_{s}^{0}$) $\overset{!}{=}$ (0.5, 0.5) while keeping the growth function parameters as defined for calculating the data plotted in Fig 3(b).

In the analysis we have so far concentrated on stem cell dynamics described by the reduced 2D version Eq (11) of the 3D system defined by Eqs (4a), (4c) and (4e), which corresponds to model A without differentiated cell compartment and neglect of mutations.

The lack of consideration of clone *x*_*s*1_(*t*) in Eq (11) is motivated by the numerical observation that with a parameterization according to Table 2, the *x*_*s*1_(*t*) population disappears asymptotically. Ref. [34] shows that it is actually a practical procedure for an investigation of global dynamics to break down an *n*-dimensional equation system composed of subequations of analog structure into blocks of lower dimensionality. Based on a comparison of a modular ‘series’ of 2D and 3D equation systems, Ref. [34] concludes that the dynamical properties of an *n*-1-dimensional stem cell model are preserved in an extended *n*-dimensional system, i.e., equilibria and bifurcation structures of the *n*-1-dimensional subsystem are mapped on the faces of an *n*-dimensional hypercube. The types of connected and isolated equilibria of the *n*-dimensional model comply with those of the *n*-1-dimensional subsystem.

Of particular relevance is the bifurcation analysis of the 3D model C of Ref. [34] which can be directly translated to stem cell dynamics described by Eqs (4a), (4c) and (4e) (for *ν*_2_ ≔ 0.0 and *ν*_3_ ≔ 0.0). Two additional equilibrium structures, a mixed-species equilibrium and a co-dimension one surface of equilibria, have been identified for model C of Ref. [34] as compared to the 2D model A. This means that a total of 13 equilibrium structures can be attributed to the solutions of 3D equation systems like model C of Ref. [34] or of the system formed from Eqs (4a), (4c) and (4e) (cf. Theorem 3 of Ref. [34]).

Refs. [33, 34] generally define 2D, 3D and 4D systems describing competition between normal and leukemic stem cell clones, partly including quiescent stem cell growth environments in the analysis. Considered in terms of systems of this type, therapy aims to drive the long term solutions towards single-state equilibria representing a stem cell niche entirely populated by healthy cells. Refs. [33, 34] show that the property of bifurcation coefficients $\gamma_{i}≔\frac{\alpha_{i}-\delta_{i}}{\delta_{i}}$ and $\gamma_{j}≔\frac{\alpha_{j}-\delta_{j}}{\delta_{j}}$, defined for each function representing the cycling state of normal clone *i* and of leukemic clone *j*, respectively, to facilitate the formulation of criteria for a differentiation between various equilibria (cf. Fig 11.6 of Ref. [33] and Fig 4 of Ref. [34]) can be utilized to predict the effect of drug administration. The parameters *α*_*j*_ and *δ*_*j*_ are of key importance in this context as they constitute the two main “levers” a therapy can adjust: cytotoxic approaches will mostly increase *δ*_*j*_, while targeted TKIs may predominantly reduce *α*_*j*_, depending on the details of disease mechanisms (the *j* indexes the specific CML clones). However, the systems described by Eqs (4), (7) and (8) model the competition between different resistant CML strains *j*, and normal cells are not explicitly included in the dynamics. The only asymptotic solution of interest from a therapeutic perspective is therefore the origin equilibrium. According to Refs. [33, 34], satisfying the condition *γ*_*j*_ ≤ 0.0 corresponds to elimination of each CML clone *j*.

#### (3.2.3) Comparison to clinical data

Comparison of the fourteen PPs of Ref. [41] shows several similarities with the simulations shown in Fig 3, including the general pattern of rapid decay of the initially dominant clone, transient dominance of one or more intermediate clones, and final dominance of a single clone. For example, PP11 may be represented by associating Y235H with *x*_*d*1_(*t*), F317L with *x*_*d*2_(*t*) and T315I with *x*_*d*3_(*t*). Similarly, PP7 also shows an initial rapid decline of Y235H, appearance of F317I, and later appearance of T315I, although the growth of T315I is slower than in PP11. The total population of differentiated cells, *y*(*t*), is proportional to the total BCR-ABL1 / beta-glucuronidase (GUS) ratio, which for many patients remains roughly constant, despite the variations of the individual clones. The simulations of Fig 3 do not generally show this pattern, but instead begin with a steep decline also in the total population. The difference may reflect a greater importance of clonal competition for population dynamics under new therapy than in our simulations, which additionally disadvantages clone 1 with a higher decay rate (equivalent to lowered symmetric division rate of *x*_*s*1_(*t*)) under the new drug. An exception among the patient data is PP15, for which the dominant clone, Y253H, rapidly falls off, nearly parallel to the decline in the total burden, to be replaced by T315I as the sole leukemic clone. Despite the variation in both qualitative and quantitative patterns of the patient data, the ability of the simple model A to reproduce the general characteristics by appropriate choices of clonal competition parameterization reinforces the view that the mechanisms of clonal contest, and the way they are modulated by the presence of therapeutic drug, are key to understanding patient data of clonal dynamics.

### (3.3) Model B: Population dynamics including a linear source term (quiescent stem cells) for the x_*s1*_(t) population (Figs 4–6)

This ansatz, outlined in Sec. 2.4, explicitly includes a quiescent stem cell growth environment *x*_*q*1_(*t*) and assumes the pool is finite and decreasing with time at a rate proportional to the quiescent cell population (spontaneous activation).

**Fig 4:**
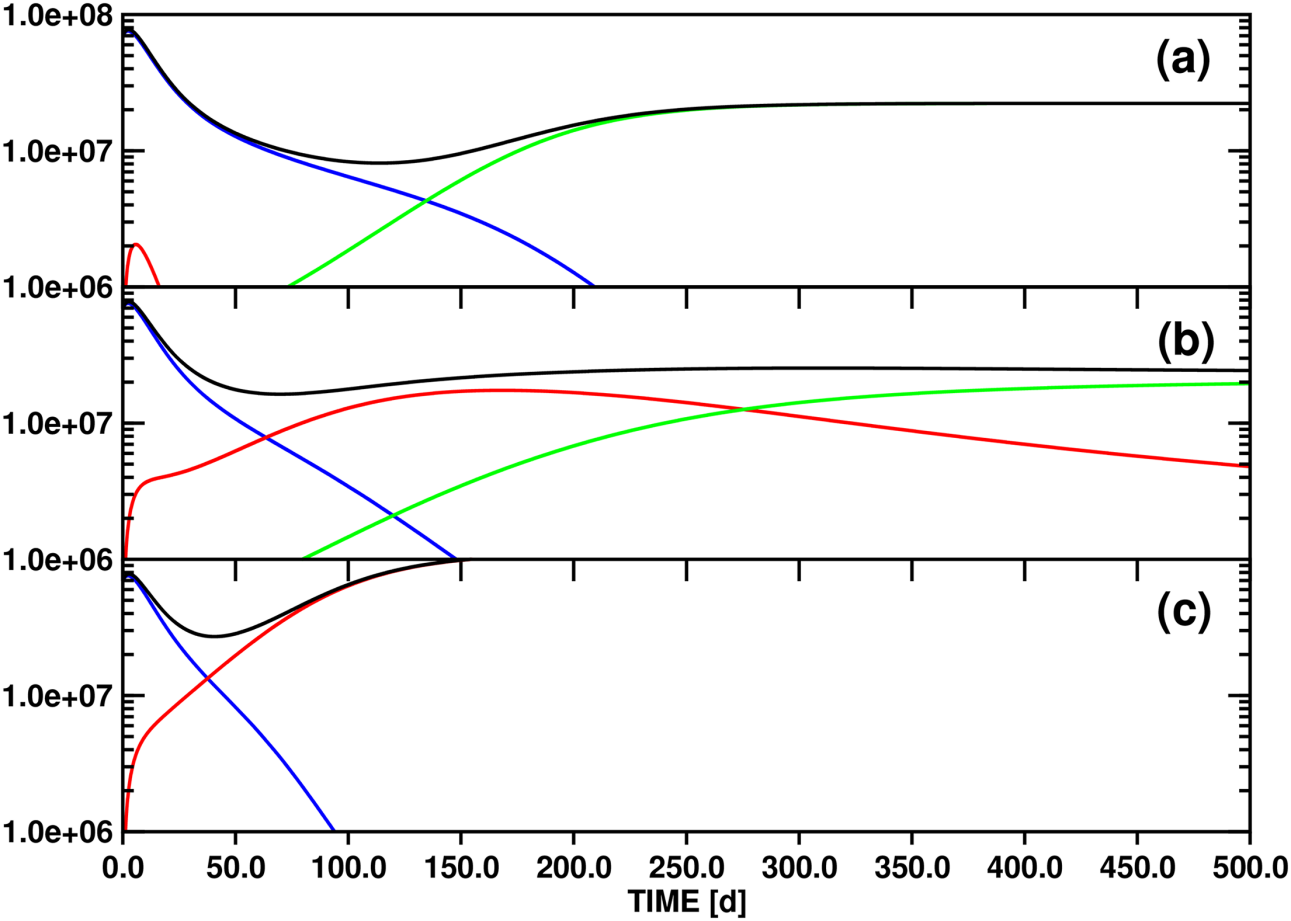
Test simulations of the population dynamics of three *imatinib*-resistant differentiated cell clones and of the total number of differentiated cells based on model B (Eq (7)) with parameters *x*_*q*1_(0) = 10.0 and *ν*_*q*1_ = 0.1. The panels (a), (b) and (c) correspond to the threshold settings *θ*_*s*2_ = 10.0, 100.0 and 500.0, respectively. The other parameters employed for the simulations are compiled in Table 2. Color scheme: *x*_*d*1_ (blue), *x*_*d*2_ (red), *x*_*d*3_ (green) and *y* (black). The ordinate unit is number of cells / ml blood.

**Fig 5:**
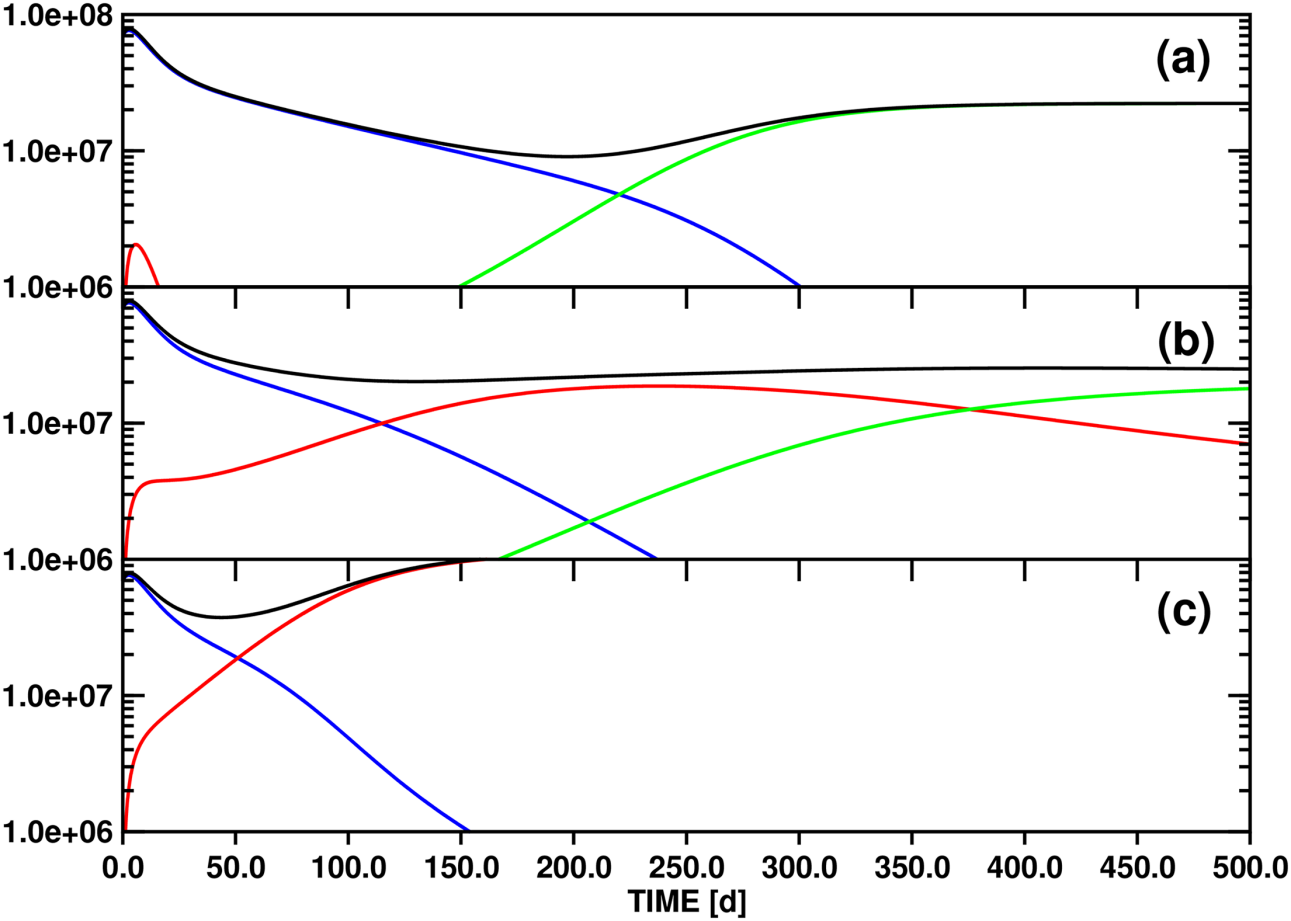
Results obtained with the same model as Fig 4, except that *x*_*q*1_(0) = 100.0.

**Fig 6:**
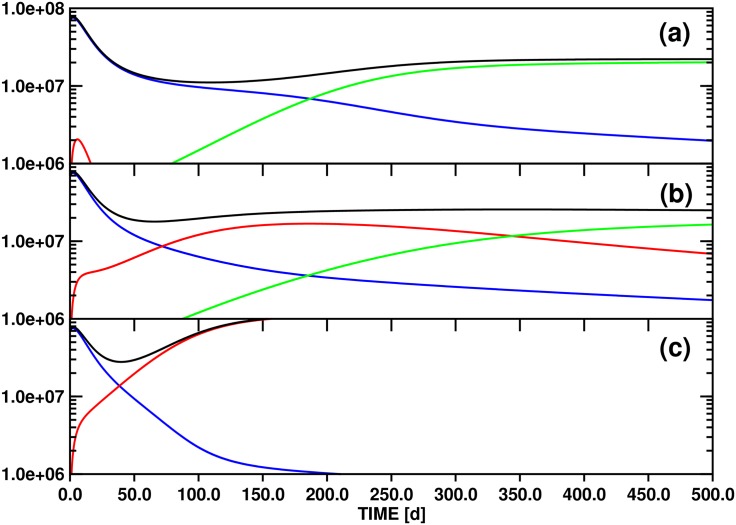
Results obtained with the same model as Fig 4, except that *x*_*q*1_(0) = 100.0 and *ν*_*q*1_ = 0.01.

The simulations illustrated in Figs 4–6, like Fig 3, compare simulations for the values *θ*_*s*2_ ≔ 10.0, 100.0 and 500.0, and for three representative *x*_*q*1_(0), *ν*_*q*1_ parameter combinations. Short initial equilibration periods of the *x*_*d*1_(*t*), *x*_*d*2_(*t*) and *x*_*d*3_(*t*) populations are observed in all simulations, similar to the scenario shown for model A in Fig 2(b). As in the case of Fig 3, the initial surge of *x*_*d*1_(*t*) towards quasi-static equilibrium occurs too rapidly (within 3 days) for a proper representation on the time scale of the figures.

With rate constant *ν*_*q*1_ ≔ 0.1 and initial population *x*_*q*1_(0) ≔ 10.0, model B shows no significant change from model A (Fig 4). The decline of *x*_*d*1_(*t*) is slightly delayed, as is the appearance of *x*_*d*3_(*t*). Expanding *x*_*q*1_(0) by a factor 10.0 to 100.0, thus increasing the resupply rate of *x*_*d*1_(*t*) (via *x*_*s*1_(*t*)) tenfold, greatly retards the decline of *x*_*d*1_(*t*) and appearance of *x*_*d*3_(*t*) (Fig 5). Reducing *ν*_*q*1_ tenfold to 0.01 while keeping *x*_*q*1_(0) ≔ 100.0 restores the initial resupply rate of *x*_*d*1_(*t*) (via *x*_*s*1_(*t*)) to the ineffective rate of the *ν*_*q*1_ ≔ 0.1, *x*_*q*1_(0) ≔ 10.0 combination (cf. Fig 4), but the two changes together also greatly defer depletion of the quiescent cell reservoir *x*_*q*1_(*t*), such that resupply continues much longer. In this case, the decay of *x*_*d*1_(*t*) is delayed to the extent that significant populations exist at the end of the simulations (500d) in both cases of *θ*_*s*2_ ≔ 10.0 (Fig 6(a)) and of *θ*_*s*2_ ≔ 100.0 (Fig 6(b)). In fact, for *x*_*q*1_(0) ≔ 100.0, *ν*_*q*1_ ≔ 0.01 is the value that maximizes the lifetime, i.e., the period until the population disappears, of the *x*_*d*1_(*t*) cell pool. For lower values, *ν*_*q*1_ *x*_*q*1_(*t*) fails to resupply fast enough to compensate the competitive reduction in asymmetric division induced by *x*_*d*3_(*t*), and for higher values, the *x*_*q*1_(*t*) reservoir is depleted too quickly for effective prolongation of the lifetime. Although maximized, the prolongation cannot alter the asymptotic behavior, as *x*_*q*1_(*t*) will inevitably become depleted with this model.

We note particularly good agreement between this model, with threshold *θ*_*s*2_ ≔ 100.0 (Fig 6(b)), and PP14, considering the clones Y253H, F317L and V299L to be represented by the functions *x*_*d*1_(*t*), *x*_*d*2_(*t*) and *x*_*d*3_(*t*), respectively.

### (3.4) Model C: Introducing population dependent transformation of stem cells from cycling to quiescence

#### (3.4.1) Mechanistic interpretation of test simulations (Figs 7–9)

**Fig 7:**
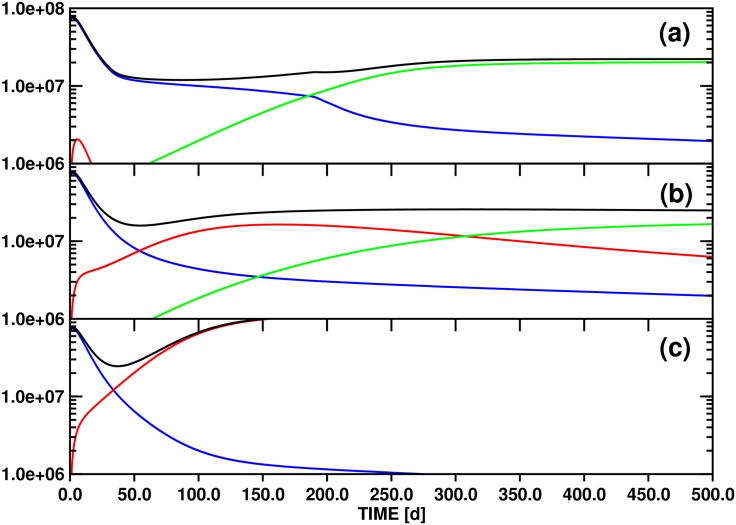
Test simulations of the population dynamics of three *imatinib*-resistant differentiated cell clones and of the total number of differentiated cells based on model C (Eq (8)) for parameters *x*_*q*1_(0) = 100.0, *ν*_*q*1_ = 0.01, *θ*_*q*1_ = 1.5 × 10^7^ and *ν*_*c*1_ = 0.1. The panels (a), (b) and (c) correspond to the threshold settings *θ*_*s*2_ = 10.0, 100.0 and 500.0, respectively. The other parameters employed for the simulations are compiled in Table 2. Color scheme: *x*_*d*1_ (blue), *x*_*d*2_ (red), *x*_*d*3_ (green) and *y* (black). The ordinate unit is number of cells / ml blood.

**Fig 8:**
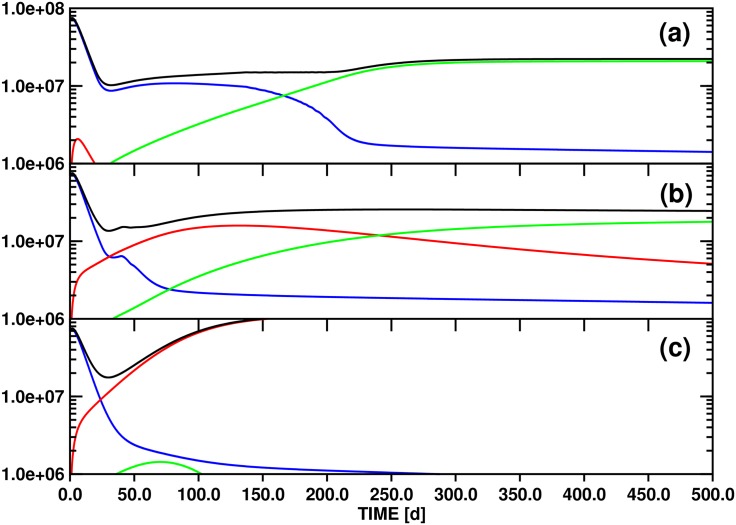
Results obtained with the same model as Fig 7, except that *ν*_*c*1_ = 0.50.

**Fig 9:**
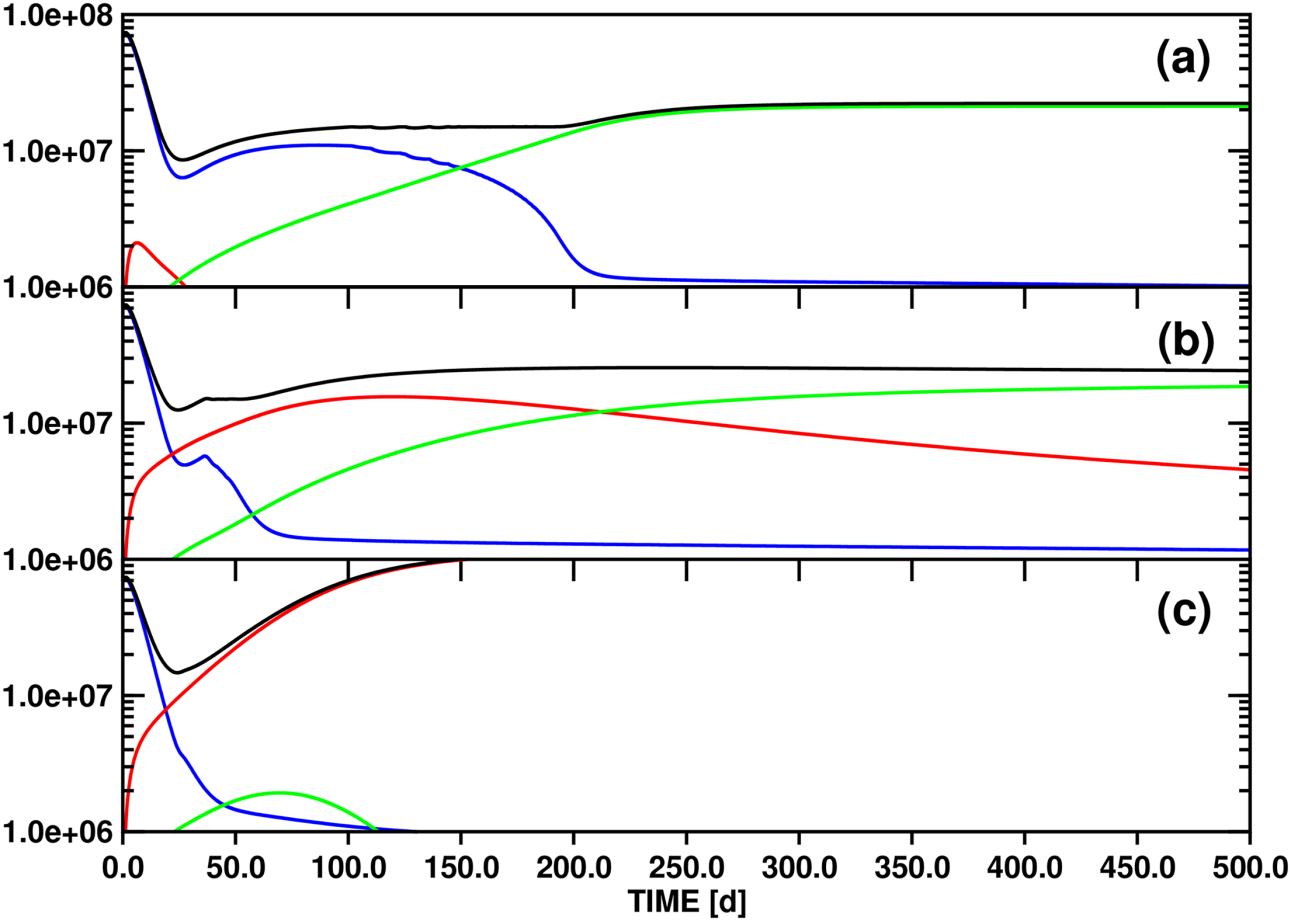
Results obtained with the same model as Fig 7, except that *ν*_*c*1_ = 1.00.

Model C introduces a population dependent transformation of cycling stem cells to quiescence, or using the model terminology, features a transfer of population from *x*_*s*1_(*t*) to *x*_*q*1_(*t*) with rate term *ν*_*c*1_ *x*_*s*1_(*t*) when the total population $y\left(t\right)=\sum_{j=1}^{3}x_{dj}\left(t\right)$ of differentiated cells surpasses threshold *θ*_*q*1_. As above, model calculations have been performed for the three values *θ*_*s*2_ ≔ 10.0, 100.0 and 500.0, and evaluated with respect to variation of the rate of transfer to quiescence, with *ν*_*c*1_ ≔ 0.1, 0.5, and 1.0, respectively (Figs 7–9). The other quiescence dynamics parameters are fixed at *x*_*q*1_(0) ≔ 100.0, *ν*_*q*1_ ≔ 0.01 and *θ*_*q*1_ ≔ 1.5 × 10^7^. The simulations are thus most directly comparable to model B (the linear source term model) and parameters as shown in Fig 6. At nearly five times the threshold to initiate quiescence, the large initial population of differentiated cells *x*_*d*1_(0) ≔ 7.0 × 10^7^ ensures that *x*_*s*1_(*t*) → *x*_*q*1_(*t*) transformation takes place from the beginning of the simulation.

The initial time evolution of the *x*_*d*1_(*t*), *x*_*d*2_(*t*) and *x*_*d*3_(*t*) clones is again characterized by rapid equilibration (as with model A, Fig 2(b)). For the smallest value, *ν*_*c*1_ ≔ 0.1, there is little qualitative difference to the corresponding simulations of model B (comparing Figs 7 and 6). At about 20d, the differentiated cell population falls to a level which switches off the *x*_*s*1_(*t*) → *x*_*q*1_(*t*) quiescence conversion for all choices of *θ*_*s*2_ (and most obviously for *θ*_*s*2_ ≔ 10.0). Until this time, *x*_*d*1_(*t*) falls more steeply with model C as the parental stem cell reservoir *x*_*s*1_(*t*) is more rapidly depleted. A second qualitative difference is the occurrence of a plateau phase that ends in a second steep population decline at approximately 200d for *θ*_*s*2_ ≔ 10.0. This later decline phase corresponds to reinitiation of the *x*_*s*1_(*t*) → *x*_*q*1_(*t*) quiescence conversion due to the growing population of the second clone, *x*_*s*2_(*t*). Thus, detailed comparisons of the plots show that the ***x*_*s*1_(*t*) → *x*_*q*1_(*t*)** quiescence transition results in a slightly more rapid decline in *x*_*s*1_(*t*) populations early in the simulation, but prolongs lifetimes later.

The five-fold larger rate of conversion to quiescence, *ν*_*c*1_ ≔ 0.5, magnifies these properties (Fig 8). Sharp features in the curves of Fig 8(a) (*θ*_*s*2_ ≔ 10.0) and Fig 8(b) (*θ*_*s*2_ ≔ 100.0) can be seen as the total differentiated cell population *y*(*t*) drops below the *θ*_*d*_ threshold at around 30d, then again at 200d or 50d, respectively, as growth of *x*_*s*2_(*t*) and/or *x*_*s*3_(*t*) reinitiates quiescence conversion. The more rapid rate also shortens the time required for the populations of *x*_*s*1_(*t*) and *x*_*q*1_(*t*) to reach equilibrium. In addition, the faster decay of *x*_*s*1_(*t*) reduces the clonal competition environment, allowing earlier growth of *x*_*s*3_(*t*) and consequently earlier appearance of *x*_*d*3_(*t*). As in the case of *ν*_*c*1_ ≔ 0.1, the total differentiated cell population *y*(*t*) never falls below the threshold *θ*_*d*_ when clone 2 is insensitive to clonal competition (*θ*_*s*2_ ≔ 500.0). But with *ν*_*c*1_ ≔ 0.5, the decline of *x*_*d*1_(*t*) is so rapid, reducing the clonal competition environment, that *x*_*d*3_(*t*) appears transiently, something not seen for models A or B at *θ*_*s*2_ ≔ 500.0.

Finally, for the largest value, *ν*_*c*1_ = 1.0, all the new features observed for model C become most pronounced (Fig 9): the initial depletion of *x*_*d*1_(*t*) is most rapid, features of halting and reinitiating the quiescence transition for *θ*_*s*2_ ≔ 10.0 and *θ*_*s*2_ ≔ 100.0 are sharpest, and, for *θ*_*s*2_ ≔ 500.0, *x*_*d*3_(*t*) appears transiently, here uniquely exceeding the population *x*_*d*1_(*t*). Persistence and recovery of the *x*_*d*1_(*t*) population is a key feature for analysing patient data and considering optimal therapeutic strategies, so we collect the effects of the various parameters on this feature in Table 4.

**Table 4:** Analysis of the solutions of model C (Eq (8)): Overview of the dependence of the formation of a *x*_*d*1_(*t*) recurrency on the individual parameters *ν*_*c*1_, *ν*_*q*1_, *θ*_*q*1_, *θ*_*s*2_ and *x*_*q*1_(0). The effects of tuning *ν*_*c*1_ and *θ*_*s*2_ on the functions of Eq (8) are also documented in Figs 7–9.

Increase of *ν*_*c*1_	Promotes development and decreases duration of *x*_*d*1_(*t*) recurrence.
Increase of *ν*_*q*1_	Promotes development and does not affect duration of *x*_*d*1_(*t*) recurrence.
	Leads to increase of asymptotic *x*_*d*1_(*t*) population.
Increase of *θ*_*q*1_	Promotes development and increases duration of *x*_*d*1_(*t*) recurrence.
Increase of *θ*_*s*2_	Reduces development and decreases duration of *x*_*d*1_(*t*) recurrence.
Increase of *x*_*q*1_(0)	Increases duration of *x*_*d*1_(*t*) recurrence. Leads to increase of asymptotic *x*_*d*1_(*t*) population.

The long term behavior of the solutions to the equations is also of importance, as it links model parameters to persistence mechanisms for individual clones. For model A, variations in the individual clone sensitivities to clonal competition determined whether there were one or two long term persistent clones, and also the relative populations of those clones. These nonzero asymptotic solutions to the equations reflect long term resistance under therapy. In contrast, the addition of quiescence to the model, and especially of signals to induce a transformation to quiescence, created longer term persistence of clones sensitive to the drug at low levels. This persistence is, however, not asymptotic, and long term therapy would ultimately deplete the quiescent pool and eliminate the clone.

The effect of the inclusion of quiescent stem cell reservoirs in models describing stem cell competition on the global dynamics of these equation systems has been investigated in Ref. [33] (models B and C) and in Ref. [34] (model B). In contrast to model C (Eq (8)), linear rate expressions have been employed to describe not only activation but also deactivation of stem cells in the respective models of Ref. [33, 34]. However, the total leukemic burden generally exceeds the threshold *θ*_*q*1_ asymptotically in the test simulations reported in this section, which means that also the population dependent term representing deactivation of *x*_*s*1_(*t*) in Eqs (8a) and (8g) becomes effectively linear in *x*_*s*1_(*t*) at the equilibria. The statement of Ref. [34], that addition of a quiescent stem cell growth environment does not qualitatively modify the deterministic bifurcation landscape of the corresponding schemes governing the dynamics of cycling stem cells, is therefore also valid within the parameterization limits for model C compiled in Table 2.

#### (3.4.2) Mechanistic interpretation of patient simulations (Figs 10–14)

PP14 of Ref. [41] provides BCR-ABL1 / GUS ratio datasets with six elements each for clones Y253H, F317L and for the total leukemic burden on an equidistant grid covering a 15 months follow-up treatment period with *dasatinib*. In addition, datasets with four elements each for the delayed clones V299_1 and V299_2 are available on the same grid, but only from month six on. No data of the BCR-ABL1 / GUS ratio for clones V299_1 and V299_2 at grid points [0, 3] are included in PP14, probably because BCR-ABL1 transcript levels in blood samples were too low in the initial *dasatinib* treatment phase. BCR-ABL1 / GUS ratio datasets with four elements each for clones Y253H, T315I and for the total leukemic burden, with values at grid points [0, 3, 9, 12], represent PP15.

**Fig 10:**
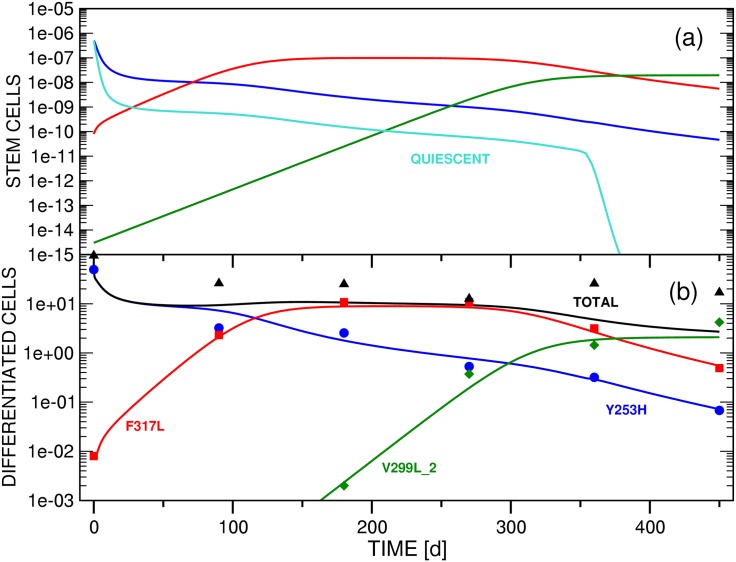
Fit to PP14 using model C, mutations of stem cells are not taken into account (*ν*_2_ = 0.0, *ν*_3_ = 0.0). The experimental values for clones Y253H (blue circles), F317L (red squares) and V299L_2 (green diamonds) are considered, clone V299L_1 is ignored by the fit. The evolution of stem and differentiated cell populations is shown in panels (a) and (b), respectively. The teal curve in panel (a) corresponds to the quiescent population of stem cell clone Y253H. In panel (b), the following identifications are made: *x*_*d*1_(*t*)(blue curve)↔Y253H, *x*_*d*2_(*t*)(red curve)↔F317L, *x*_*d*3_(*t*)(green curve)↔V299L_2. The black curve and triangles correspond to the fitted and experimental values of the total leukemic burden, respectively. The ordinate unit of panel (b) refers to the differentiated cell BCR-ABL1 / GUS ratios of the respective clones. While panel (b) reproduces also clinical datasets, the ordinate of panel (a) specifies the size of the stem cell populations in arbitrary units, the scale is determined by the parameters *a*_*i*_. The parameter set employed for this simulation is compiled in Table 3.

**Fig 11:**
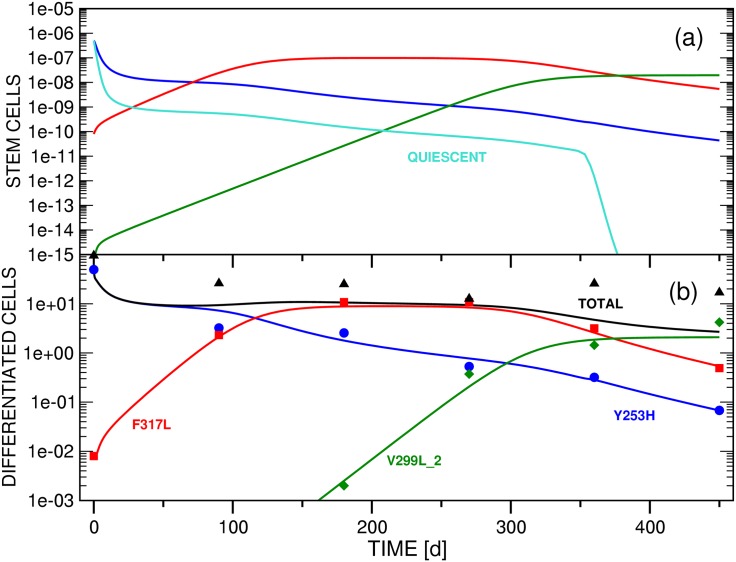
Description is the same as for Fig 10, except that mutations *x*_*s*1_(*t*) → *x*_*s*3_(*t*) are included in the simulation (*ν*_3_≠0, cf. Table 3).

**Fig 12:**
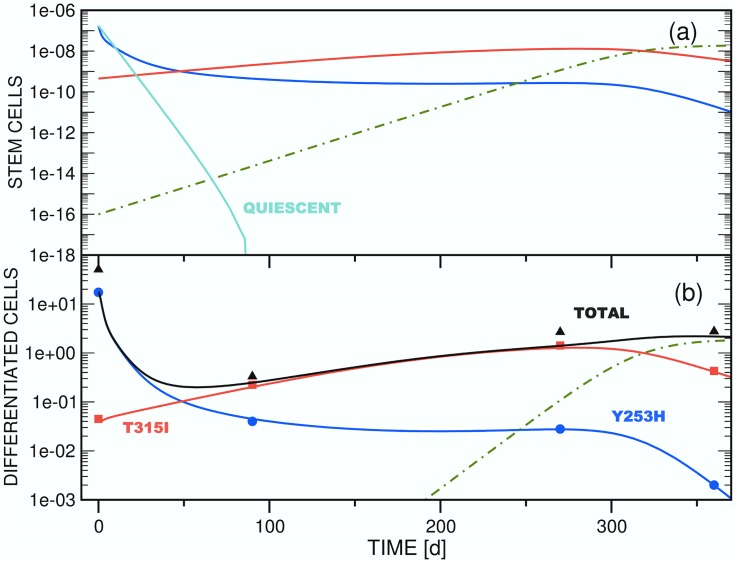
Fit to PP15 using model C, including the experimental values for clones Y253H (blue circles) and T315I (red squares). Mutations of stem cells are not taken into account (*ν*_2_ = 0.0, *ν*_3_ = 0.0). The evolution of stem and differentiated cell populations is shown in panels (a) and (b), respectively. The teal curve in panel (a) corresponds to the quiescent population of stem cell clone Y253H. In panel (b), the following identifications are made: *x*_*d*1_(*t*)(blue curve)↔Y253H, *x*_*d*2_(*t*)(red curve)↔T315I, *x*_*d*3_(*t*)(green curve)↔X. The black curve and triangles correspond to the fitted and experimental values of the total leukemic burden, respectively. The ordinate unit of panel (b) refers to the differentiated cell BCR-ABL1 / GUS ratios of the respective clones. While panel (b) reproduces also clinical datasets, the ordinate of panel (a) specifies the size of the stem cell populations in arbitrary units, the scale is determined by the parameters *a*_*i*_. The parameter set employed for this simulation is compiled in Table 3. This simulation investigates the role of a hypothetical third clone X that is not explicitly identified by experiment. The dashed-dotted green line represents stem and differentiated cell populations of this hypothetical third clone.

**Fig 13:**
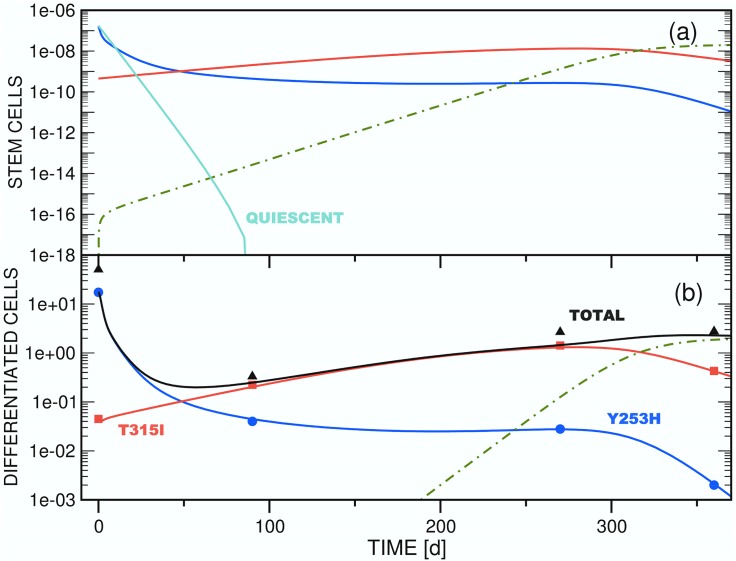
Description is the same as for Fig 12, except that mutations *x*_*s*1_(*t*) → *x*_*s*3_(*t*) are included in the simulation (*ν*_3_≠0, cf. Table 3).

**Fig 14:**
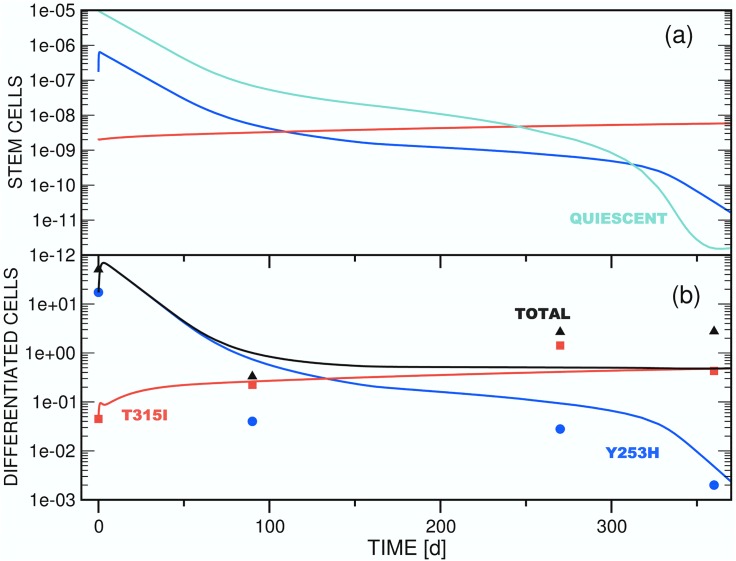
Fit to PP15 using model C (with populations *x*_*s*3_(*t*) and *x*_*d*3_(*t*) set to 0.0), including the experimental values for clones Y253H (blue circles) and T315I (red squares). Mutations of stem cells are not taken into account (*ν*_2_ = 0.0, *ν*_3_ = 0.0). The evolution of stem and differentiated cell populations is shown in panels (a) and (b), respectively. The teal curve in panel (a) corresponds to the quiescent population of stem cell clone Y253H. In panel (b), the following identifications are made: *x*_*d*1_(*t*)(blue curve)↔Y253H, *x*_*d*2_(*t*)(red curve)↔T315I. The black curve and triangles correspond to the fitted and experimental values of the total leukemic burden, respectively. The ordinate unit of panel (b) refers to the differentiated cell BCR-ABL1 / GUS ratios of the respective clones. While panel (b) reproduces also clinical datasets, the ordinate of panel (a) specifies the size of the stem cell populations in arbitrary units, the scale is determined by the parameters *a*_*i*_. The parameter set employed for this simulation is compiled in Table 3.

In the simulations shown in Figs 10–13, all parameters of model C (cf. Table 3) have been varied using a least squares fitting algorithm based on the datasets PP14 and PP15 (not including the observed total leukemic burden values). Fig 14 is, however, an exception, since only parameters relating to clones Y253H and T315I, i.e., to functions *x*_*s*1_(*t*), *x*_*s*2_(*t*), *x*_*d*1_(*t*) and *x*_*d*2_(*t*) of model C, have been included in the fitting procedure.

Fig 10 illustrates the result of a fit to the datasets of clones Y253H (*x*_*d*1_(*t*)), F317L (*x*_*d*2_(*t*)) and V299_2 (*x*_*d*3_(*t*)) of PP14 for model C. Clone V299_1 has been ignored in the fitting routine. The simulation assumes that mutations of the wild-type CML clone into the *imatinib*-resistant clones Y253H, F317L and V299_2 are limited to the period of *imatinib* treatment by defining nonzero initial stem cell populations *x*_*s*1_(0), *x*_*s*2_(0) and *x*_*s*3_(0). While *x*_*d*1_(0) and *x*_*d*2_(0) can be taken directly from PP14, *x*_*d*3_(0) is not known experimentally but is assumed to be below the detection limit and set to zero. Since also no clinical information on the initial population of quiescent stem cells of clone Y253H is available, the value of *x*_*s*1_(0) has been adopted also for *x*_*q*1_(0).

Panels (a) and (b) of Fig 10 show that each clone pair of stem and differentiated cell populations remains proportional (cf. Eq (10)). In contrast to the test simulations, no rapid initial equilibration with *x*_*s*1_(*t*) is observed, and instead, *x*_*si*_(*t*) / *x*_*di*_(*t*) (*i* = 1, 2, 3) proportionality is present from the beginning. The *x*_*s*1_(*t*) / *x*_*d*1_(*t*) cell pools are characterized by rapid decay within the first ca. 20d in response to *dasatinib* application. The early depletion phase is followed by a stabilization of *x*_*s*1_(*t*) / *x*_*d*1_(*t*) up to ca. 90d, subsequent exponential decay until ca. 150d, although not as rapid as in phase 1, a second stabilization period until ca. 300d, which is less pronounced than the first one, and finally again exponential decay towards the 450d mark at an only slightly reduced rate as compared to phase 2.

Inspection of Fig 10(b) shows that *y*(*t*) = ∑_*j*_ *x*_*dj*_(*t*) > *θ*_*q*1_ in the initial 350d period, which implies that activation and deactivation of stem cells occur in quasi-static equilibrium. However, because *x*_*s*1_(0) = *x*_*q*1_(0), the large ratio *ν*_*q*1_ / *ν*_*c*1_ = 16.84 leads to an equilibration of the quiescent and cycling stem cells within ca. 10d. As a result, the *x*_*q*1_(*t*) pool drains quicker than *x*_*s*1_(*t*) in this starting phase until the balance of the activation and deactivation rates is reached, a state distinguished by the parallel development of the *x*_*s*1_(*t*) and *x*_*q*1_(*t*) populations towards the 350d mark. A critical limit is encountered just beyond 350d: ∑_*j*_ *x*_*dj*_(*t*) falls below the threshold *θ*_*q*1_ = 5.0 and *x*_*s*1_(*t*)-deactivation is switched off from this point on. Consequently, the *x*_*s*1_(*t*)-*x*_*q*1_(*t*) balance is destroyed and the *x*_*q*1_(*t*) population drops exponentially within ca. 10d. The rapid disappearance of the quiescent growth environment does not have a significant effect on *x*_*s*1_(*t*), however, since the value *x*_*q*1_(350) is already quite low.

The growth of the *x*_*s*2_(*t*) and *x*_*d*2_(*t*) populations until ca. 100d can be divided into a very fast (until ca. 10d) and a slightly slower exponential phase. The *x*_*s*2_(*t*) / *x*_*d*2_(*t*) cell pools subsequently consolidate and dominate the leukemic burden until ca. 300d are reached.

Beyond 300d, competition from strain *x*_*s*3_(*t*) becomes evident, with parallel exponential decay of *x*_*s*2_(*t*) and *x*_*d*2_(*t*) afterwards. Clone *x*_*s*3_(*t*) / *x*_*d*3_(*t*) is characterized by an extended exponential growth period starting from a very low stem cell level and continuing for ca. 300d before transition into an asymptotic saturation phase. The *x*_*s*3_(*t*) / *x*_*d*3_(*t*) populations supplant *x*_*s*2_(*t*) / *x*_*d*2_(*t*) as the dominant clone by 390d.

Fig 10(b) shows that the fitted curves *x*_*d*1_(*t*), *x*_*d*2_(*t*) and *x*_*d*3_(*t*) represent a good approximation to the clinical datasets for clones Y253H, F317L and V299_2, respectively.

The coefficients of determination *R*^2^ for *x*_*d*1_(*t*), *x*_*d*2_(*t*) and *x*_*d*3_(*t*) are provided in Table 5. The fact that the experimental total leukemic burden (black triangles) exceeds *y*(*t*) (black curve) substantially except for the values at 270d is consistent with PP14 and may reflect unidentified BCR-ABL1 clone(s), e.g., contributions from the wild-type CML strain. In the case of the grid points 360d and 450d, however, a part of the relatively large deviation between experimental and theoretical total leukemic burden can be attributed to omission of clone V299_1 from the simulation as the observed population of this clone is substantial at 360d and 450d (cf. PP14).

**Table 5:** Assessment of the quality of the fit of the theoretical population function *x*_*di*_ for clone *i* to a clinical differentiated CML cell population data set (${\tilde{x}}_{di_{1}},{\tilde{x}}_{di_{2}},\cdots$). The fitting method is described in Sec. 2.2.3. This table provides the coefficient of determination *R*^2^ for each individual fit curve *x*_*di*_(*t*) shown in panel (b) of Figs 10–14. Note that the black curves representing the theoretical total leukemic burden *y*(*t*) in each figure have not been included in the fitting procedure but have been obtained by simply adding the calculated populations of the differentiated cell clones *x*_*di*_(*t*) at each time step. Therefore, no *R*^2^ coefficients are given for the total leukemic burden curves. *R*^2^ is defined as $\sum_{k}{\left(x_{di_{k}}-{\bar{x}}_{di}\right)}^{2}/{\left({\tilde{x}}_{di_{k}}-{\bar{x}}_{di}\right)}^{2}$, where ${\bar{x}}_{di}$ is the mean of the observed values ${\tilde{x}}_{di_{k}}$ and the summation index *k* refers to the time values *t*_*k*_ at which the ${\tilde{x}}_{di_{k}}$ have been determined. The closer the value of *R*^2^ is to 1.0, the better is the fit approximation.

Clone (color)	Fig 10(b)	Fig 11(b)	Fig 12(b)	Fig 13(b)	Fig 14(b)
blue	0.9766	0.9768	0.9998	0.9998	0.9710
red	1.0565	1.0583	1.0330	1.0255	1.1894
green	0.8602	0.8576	-	-	-

The simulations displayed in Figs 10 and 11 look very similar and the pertinent parameterizations of model C differ only with respect to *ν*_3_ and *x*_*s*3_(0) (cf. Table 3). According to PP14, strain V299_2 appears only after six months of treatment with *dasatinib*. While the scenario investigated by employing the parameter set of Fig 10 assumes that a small population *x*_*s*3_(0) of V299_2 stem cells has developed during previous *imatinib* administration, a very small rate constant *ν*_3_ is driving *x*_*s*1_(*t*) → *x*_*s*3_(*t*) mutations in the simulation shown in Fig 11.

With respect to the accuracy of the *x*_*d*1_(*t*), *x*_*d*2_(*t*) and *x*_*d*3_(*t*) fits, the *R*^2^ coefficients of Table 5 prove that the quality of the fit approximations of Fig 11(b) is only slightly lower as compared to the fits of Fig 10(b). Figs 10 and 11 differ mainly in the course of the *x*_*s*3_(*t*) population within the [0d, 5d] interval. The otherwise good agreement between both simulations demonstrates that it is not possible, based on the sparse clinical data set, to decide whether a nonzero initial population of V299_2 stem cells at the beginning of *dasatinib* medication or a low Y253H→V299_2 mutation rate during *dasatinib* therapy or possibly both factors together are responsible for the delayed appearance of clone V299_2 in PP14. Obviously, it would be of great interest to obtain experimental information on the population of V299_2 stem cells shortly before the *dasatinib* application period.

PP15 has a profile comprising only two competing CML strains, Y253H and T315I. Fig 14 shows model C as fit to PP15 data, whereby *x*_*s*1_(*t*) / *x*_*d*1_(*t*) and *x*_*s*2_(*t*) / *x*_*d*2_(*t*) represent Y253H and T315I, respectively. In this case, model C is reduced from a 7D to a 5D model by setting *x*_*s*3_(*t*) and *x*_*d*3_(*t*) to zero. Mutations are supposed to be absent (*ν*_2_ ≔ 0, *ν*_3_ ≔ 0). The clones Y253H and T315I originated during the preceding phase of *imatinib* treatment and the starting populations *x*_*d*1_(0) and *x*_*d*2_(0), respectively, are provided by PP15.

For the initially dominant clone Y253H, the small ratio *ν*_*q*1_ / *ν*_*c*1_ = 0.083 favors deactivation of *x*_*s*1_(*t*) over activation of *x*_*q*1_(*t*), but since the starting size *x*_*q*1_(0) of the quiescent stem cell reservoir strongly exceeds that of the active stem cell pool (*x*_*s*1_(0), cf. Table 3), an extremely rapid (within the interval [0d, 1d]) surge of the *x*_*s*1_(*t*) population is obtained before balance with *x*_*q*1_(*t*) is established (Fig 14(a)). The sudden increase of the active stem cell branch is passed on, with a slight time delay, to the dependent differentiated cells *x*_*d*1_(*t*) (Fig 14(b)).

This combination of an early spike followed by exponential decay of the *x*_*d*1_(*t*) reservoir is reminiscent of the pattern appearing also in the test simulations (cf. Fig 2(b)). However, the source of this phenomenon in the test simulations is the definition of slightly off-balance initial population values *x*_*s*1_(0) and *x*_*d*1_(0), i.e., the *x*_*s*1_(*t*) pool undergoes immediate reduction without a weak and short-lived preceding population growth.

The rapidly shaped maximum of the *x*_*d*1_(*t*) curve seen in Fig 14(b) is, on the other hand, not primarily related to a *x*_*s*1_(*t*)–*x*_*d*1_(*t*) equilibration process but rather represents the reaction to the even swifter emergence of a vertex at the level of cycling stem cells (Fig 14(a)). The nearly instantaneous switch from a steeply rising to a decreasing *x*_*s*1_(*t*) population in turn reflects an equilibration phase induced by very differently sized initial values *x*_*s*1_(0) and *x*_*q*1_(0). Such a large *x*_*q*1_(0) / *x*_*s*1_(0) ratio is required to ensure a proper timing of the transition to an accelerated decay phase of the *x*_*d*1_(*t*) population at ca. 320d, which correlates with the onset of the *y*(*t*)<*θ*_*q*1_ regime at 235d (see below). Since *x*_*q*1_(0) is not directly known from experiment, the *x*_*q*1_(0) / *x*_*s*1_(0) ratio has been adjusted to meet this criterion.

Fig 14(a) shows that after rapid alignment, the *x*_*s*1_(*t*) and *x*_*q*1_(*t*) populations remain in a balance for 235d, before the size of the *x*_*q*1_(*t*) pool decreases progressively and more rapidly than the *x*_*s*1_(*t*) reservoir until ca. 350d. The cycling / non-cycling decay rates are reversed only in the final [350d, 370d] interval: the *x*_*q*1_(*t*) and *x*_*s*1_(*t*) curves stabilize at a very low level and decline exponentially, respectively.

We will now analyze the mechanism responsible for this relation between the *x*_*q*1_(*t*) and *x*_*s*1_(*t*) stem cell pools. As in the case of the simulations of PP14, the total leukemic burden *y*(*t*) surpasses *θ*_*q*1_ at the beginning of the calculation. Therefore, deactivation of *x*_*s*1_(*t*) is initially proceeding in addition to activation of *x*_*q*1_(*t*), however, the net effect is transfer of population from the non-cycling to the cycling growth environment since *ν*_*c*1_ *x*_*s*1_(0) = 8.3 × 10^−7^ < *ν*_*q*1_ *x*_*q*1_(0) = 4.00 × 10^−6^. From a mechanistic perspective, two situations critical for the *x*_*s*1_(*t*)–*x*_*q*1_(*t*) balance are encountered as the system evolves: (i) At 162d, activation and deactivation rates cancel: *ν*_*c*1_ *x*_*s*1_(162) = *ν*_*q*1_ *x*_*q*1_(162) = 7.10 × 10^−9^. From this point on, *ν*_*c*1_ *x*_*s*1_(*t*) > *ν*_*q*1_ *x*_*q*1_(*t*). However, a net population shift from cycling to non-cycling stem cell pool takes place only from 162d until the next mark is reached. (ii) At 235d, *y*(*t*) = *θ*_*q*1_ = 0.51 and from this instant until 370d the inequality *y*(*t*)<*θ*_*q*1_ holds. This condition implies that the deactivation term is turned off in Eqs (8a) and (8g) and therefore the *x*_*s*1_(*t*) cell pool is bolstered again from 235d on at the expense of *x*_*q*1_(*t*). The smooth transition from a net *x*_*q*1_(*t*) → *x*_*s*1_(*t*) to a net *x*_*s*1_(*t*) → *x*_*q*1_(*t*) transfer regime at 162d thus ensures the nearly synchronous, biphasic decay of the *x*_*s*1_(*t*) and *x*_*q*1_(*t*) populations stretching from the end of the initial *x*_*s*1_(*t*)–*x*_*q*1_(*t*) equilibration phase to 235d.

Fig 14(b) shows that while the agreement between the clinical values (red squares) and the model prediction (red curve, *x*_*d*2_(*t*)) is particularly poor for clone T315I at 270d, the model for Y253H (blue curve, *x*_*d*1_(*t*)) does not fit experiment (blue circles) well at all four sampling times, although the plateau and final steep decline are qualitatively reproduced (the function *x*_*d*1_(*t*) exhibits a triphasic decay, cf. Table 1). Inspection of Table 5 reveals that the runaway value at 270d substantially spoils the *R*^2^ coefficient of *x*_*d*2_(*t*) due to the large difference between ${\tilde{x}}_{d2}$(270d) and *x*_*d*2_(270d). As a result, the fit *x*_*d*1_(*t*) is more precise than *x*_*d*2_(*t*). In addition to the low accuracy, this two-clone model fit suffers from early population growth features not corroborated by the data.

Despite their similar appearance, the mechanisms for the fast preliminary expansions of the *x*_*d*1_(*t*) and *x*_*d*2_(*t*) population functions are different. The growth of *x*_*d*1_(*t*) is induced by widely divergent values of *x*_*s*1_(0) and *x*_*q*1_(0), leading to a nearly parallel time evolution of the *x*_*s*1_(*t*) and *x*_*d*1_(*t*) functions from the beginning (cf. analysis above). This is obviously not the case for the pair *x*_*s*2_(*t*) / *x*_*d*2_(*t*): model C does not include a quiescent stem cell reservoir associated with the active clone *x*_*s*2_(*t*), and the surge of *x*_*d*2_(*t*) instead represents a *x*_*s*2_(*t*)–*x*_*d*2_(*t*) equilibration process caused by an imbalance between the initial values *x*_*s*2_(0) and *x*_*d*2_(0).

If this proposed mechanism responsible for the initial steep increase of function *x*_*s*1_(*t*) would properly describe the biological system, then application of *dasatinib* would disturb the *x*_*s*1_(*t*)–*x*_*q*1_(*t*) balance by reducing the *x*_*s*1_(*t*) (and *x*_*d*1_(*t*)) populations which in response would trigger a sudden *x*_*q*1_(*t*) → *x*_*s*1_(*t*) activation. Due to the scarcity of the clinical data, a rapid growth of the Y253H and T315I populations as a reaction to the onset of *dasatinib* administration is not explicitly supported by the observed patient profile. However, the lack of experimental information in the critical time interval also does not allow to entirely exclude such a phenomenon. In fact, Ref. [51] reports a stimulatory effect of *dasatinib* on proliferation of certain clones of acute myelogenous leukemia progenitor cells *in vitro* at low dose.

However, the non-quantitative nature of the *x*_*d*1_(*t*) (Y253H) and *x*_*d*2_(*t*) (T215I) approximations to the observed data points and the necessity to define starting conditions that lead to rapid equilibration processes accompanied by somewhat artificial sudden population increases indicate that the two-clone version of model C is not appropriate for a realistic simulation of PP15.

Alternatives to the two-clone scenario are therefore of interest. For PP15, the experimentally determined total leukemic burden differs significantly from the sum of the levels of clones Y253H and T315I at the final data point at 360d, despite good agreement at 0d, 90d and 270d (Fig 14(b)). If this anomaly is not a sampling error, it may indicate the expansion of an unidentified third CML clone, consistent with other observations of delayed appearances of resistant CML clones [41] (cf. strains V299_1 and V299_2 in PP14). Should a third clone in fact be involved in the CML dynamics shaping PP15, then the question arises whether clone 3 stem cells originated (i) already during the preceding *imatinib* treatment period or (ii) in the course of *dasatinib* administration. The simulations presented in Figs 12 and 13 assume option (i) and (ii), respectively.

We first simulate PP15 employing a full 7D model C (including three CML strains Y253H (*x*_*s*1_(*t*), *x*_*q*1_(*t*), *x*_*d*1_(*t*)), T315I (*x*_*s*2_(*t*), *x*_*d*2_(*t*)) and a hypothetical third clone (*x*_*s*3_(*t*), *x*_*d*3_(*t*))). The time propagation of this three-clone ansatz yields Fig 12. Since the best fitting result was achieved by defining *ν*_*c*1_ ≔ 0.0, model C is reduced to the linear source term model (model B). The starting conditions assume that no differentiated cells of clone 3 (*x*_*d*3_(0) ≔ 0.0) are present but a very low population of cycling stem cells (*x*_*s*3_(0) ≔ 1.0 × 10^−16^). Mutations are not taken into account (*ν*_2_ ≔ 0.0, *ν*_3_ ≔ 0.0).

The initial populations *x*_*s*1_(0) and *x*_*q*1_(0) are assumed to be identical. Deactivation of *x*_*s*1_(*t*) is switched off from the beginning, resulting in rapid exponential decay of function *x*_*q*1_(*t*) until the quiescent stem cell pool is exhausted after ca. 85d (Fig 12(a)). The complete transfer of population from the quiescent to the active growth environment of clone 1 within the [0d, 85d] interval results in an efficient buffering of the sudden drop of function *x*_*s*1_(*t*), marked by a clearly distinguishable kink in the *x*_*s*1_(*t*) curve after ca. 5d. The first depletion phase is followed by a drawn-out period of non-exponential, remittent decline of the *x*_*s*1_(*t*) reservoir down to a value of *x*_*s*1_(198) = 2.5 × 10^−10^. Beyond 198d, *x*_*s*1_(*t*) interestingly passes over into an incremental mode (induced by *ω*_12_ = -0.03) before at 268d turning to a final decrease.

The *x*_*s*2_(*t*) population increases monotonically, takes over as dominant clone and reaches a maximum after 50d and 281d, respectively. The subsequent cutback of the *x*_*s*2_(*t*) reservoir is due to competition from *x*_*s*3_(*t*). The imaginary clone 3 grows exponentially, overtakes the leading *x*_*s*2_(*t*) pool in size by 315d and subsequently saturates.

Fig 12(b) proves that the dynamics in the stem cell compartment is largely reproduced in the realm of differentiated cells. A comparison with Fig 14(b) demonstrates that the three-clone ansatz yields a much better approximation to the clinical datasets of clones Y253H, T315I and of the total leukemic burden than the two-clone approach. This impression is confirmed by the *R*^2^ coefficients compiled in Table 5.

Finally, we investigate option (ii), the mutation-induced appearance of clone 3 during *dasatinib* therapy. Model C is again reduced to model B by specifying *ν*_*c*1_ ≔ 0.0 for the preparation of Fig 13. In general, the parameterizations of the simulations underlying Figs 12 and 13 differ only slightly, most notably, the mutation scenario of Fig 13 is realized via the definitions *x*_*s*3_(0) ≔ 0.0 and *ν*_3_ ≔ 3.0 × 10^−10^ (cf. Table 3). A comparison of Figs 12 and 13 confirms the good general consistency of the results. For the differentiated cell compartments, very similar *R*^2^ coefficients (cf. Table 5) of the *x*_*d*1_(*t*) (Y253H) and *x*_*d*2_(*t*) (T315I) fits shown in Figs 12(b) and 13(b) support the concordant quality of the approximations to the datasets of clones Y253H and T315I. Also the total leukemic burden (black triangles) is again well reproduced in Fig 13(b). The most prominent difference is the initialization phase of the clone 3 stem cells, with a nonzero starting value *x*_*s*3_(0) in Fig 12(a) and an immediate population build up via *x*_*s*1_(*t*) → *x*_*s*3_(*t*) mutations in Fig 13(a).

Figs 10 and 11 for PP14 as well as Figs 12 and 13 for PP15 therefore indicate that the dynamics of either third clone of these two patients may be fit well using a mutation rate instead of initial stem cell population so the model is unable to distinguish between the appearance of new resistant clones via low-level pre-existing populations or via mutation.

## Discussion

In this manuscript, we have analysed the behavior of three models of CML dynamics in patients who have been placed under new treatment after diagnosis of resistance to *imatinib*. This study represents the first theoretical investigation of competition dynamics of *imatinib*-resistant CML strains exposed to second-line medication (*dasatinib*, *nilotinib*) as observed in recent clinical trials [40, 41]. We compared the relative effects of mutation rates, initial populations, clonal competition (with differential sensitivities), and stem cell quiescence to data sets provided in Ref. [41]. In creating the models, we set out to investigate how patient data may be explained, and conversely, how the data may constrain model parameters and provide evidence for mechanisms of disease and drug action. Patterns of dynamics particularly suited for this include the differentiation between rapidly appearing vs. persistent resistant clones, and also include population declines of therapy sensitive clones that are biphasic or that proceed through a plateau or partial recovery phase. As described above, these phenomena can be explained by the models: sequential dominance by the inclusion of clonal competition with differential sensitivities (coupled to choices of initial population values), rapid clearance by stem cell net decay rate dependence on its sensitivity to drug, and biphasic or more complex decline dynamics by the inclusion of quiescence in the model.

The condition for general decline of the primary (i.e. dominant at the beginning of *dasatinib* or *nilotinib* therapy) stem cell clone 1 is *ζ*_1_(**x**_*s*_(*t*))*α*_1_ < *δ*_1_ (cf. Eq (1)), that is, the decay rate exceeds the feedback controlled symmetric division rate. Detailed modelling of the effect of the drug would require semi-quantitative knowledge of the action of aberrant BCR-ABL1 signalling on proliferation processes, as well as of drug influence across the signalling networks. In the test simulations (cf. Secs. 3.2.1, 3.3 and 3.4.1), the relative sensitivity of clone 1 (*x*_*s*1_(t)) is accomplished numerically by providing the secondary (*x*_*s*2_(t)) and tertiary (*x*_*s*3_(t)) stem cell variants with a lower decay rate (cf. Table 2), such that clonal competition eliminates *x*_*s*1_(t). The rapid initial decay of clone 1 differentiated cells *x*_*d*1_(t) in our models results from the same process, as the populations of stem (*x*_*si*_(t)) and differentiated cells (*x*_*di*_(t)) of clone *i* stay in quasi-balance, except for short equilibration phases that may be induced by biased definitions of initial populations *x*_*si*_(0) and *x*_*di*_(0) (cf. Sec. 3.2.1). As a result, the models employed for the test (and patient) simulations describe the rapid initial decay of the population *x*_*d*1_(t) frequently observed in the clinical data by assuming that the drug (*dasatinib* or *nilotinib*) downregulates asymmetric division of the parent stem cell clone (*x*_*s*1_(t)), which under the previous treatment regime (*imatinib*) was at a higher level.

For stem cells of the secondary (*x*_*s*2_(t)) and tertiary clones (*x*_*s*3_(t)), the growth and decay rates remain roughly equal, *ζ*_*i*_(**x**_*s*_(*t*))*α*_*i*_ ≈ *δ*_*i*_ (*i* = 2, 3), with the clonal competition term (via *ζ*_*i*_(**x**_*s*_(*t*))) lowering the symmetric division rate sufficiently to converge to a finite limit (zero or nonzero) asymptotically. The long term behavior depends on the parameter regimes and details of the clonal competition terms, as described for model A in Sec. 3.2.2. Persistence of resistant clones at stable levels of total leukemic burden is characteristic of most patients, with only PP8 and PP15 representing significant deviations from this pattern [41].

The test simulations show that the increasing complexity from model A to C enables progressively successful reproduction of complex patterns observed clinically (cf. Table 1). Model A can describe the rapid decay of an initially dominant clone, according to Ref. [41] frequently Y253H or M315T, and also the appearance of new *dasatinib*- or *nilotinib*-resistant strains, e.g. F317L, V299L, and T315I, as observed in PP11, PP14 or PP15. The persistence of resistant strains (especially T315I as in PP15) can also be reproduced. This model does not, however, replicate stabilization and eventual reduction of some strains, e.g. of the Y253H population reported in PP14 and PP15.

The addition of a depleting pool of quiescent stem cells (*x*_*q*1_(*t*)) that proportionally provides cycling stem cells (*x*_*s*1_(*t*)) to Eq (4) yields model B and improves the approximation of temporary stabilization of declining species, such as the multiphasic decline of the Y253H cell pool shown in PP14 and PP15. However, because in model B both the function *x*_*s*1_(*t*) and the *x*_*q*1_(*t*) → *x*_*s*1_(*t*) conversion rate must decrease monotonically over time (with a parameterization according to Table 2, which implies *ω*_*ii*_, *ω*_*ij*_ ≥ 0.0, this assumption is true for *x*_*s*1_(*t*) since the asymptotic validity of the inequality *ζ*_1_(**x**_*s*_(*t*))*α*_1_ < *δ*_1_ is ensured by a large, stable value of ∑_*i*_ *x*_*si*_), the model cannot reproduce recovery of *x*_*s*1_(*t*) by resupply from reservoir *x*_*q*1_(*t*). Instead, as described in Ref. [28], physiological and computational considerations argue for bidirectional switching between cycling and quiescent states of stem cells, that is, relative rates of activation of quiescent stem cells of clone *i* (*x*_*qi*_(*t*)) should be regulated by the size of the *x*_*si*_(*t*) population. In the model of Ref. [28], the rate terms describing activation of *x*_*qi*_(*t*) and deactivation of *x*_*si*_(*t*) are linear in *x*_*qi*_(*t*) and *x*_*si*_(*t*), respectively, and it is assumed that feedback relations exist only between the cycling and non-cycling growth environments of a single clone *i*, i.e., the cycling population *x*_*sj*_(*t*) of a competing clone *j* does not influence the net activation rate of *x*_*qi*_(*t*).

In model C a modified approach is implemented based on the idea that it would be more realistic if (i) the total population of clones *i*, *j* controls the net activation rate of *x*_*qi*_(*t*) (because the cycling stem cell populations *x*_*si*_(*t*) and *x*_*sj*_(*t*) are competing for resources in the same bone marrow niche), and (ii) a sigmoidal signalling function, or its Heaviside-type abstraction, is employed rather than a less realistic linear feedback term. More precisely, the Heaviside signalling function for the deactivation of *x*_*s*1_(*t*) depends on the total population of differentiated cells *y*(*t*) (Eq (8)), but this is only a minor detail since the sum of stem cell populations ∑_*i*_ *x*_*si*_ should be nearly proportional to *y*(*t*) according to Eq (10).

Models that are suitable for CML dynamics studies may be grouped into two categories: stochastic models are based on probabilities of fundamental cellular processes such as cell division [20, 21, 25, 52–57, 57–64], while deterministic concepts represent the time evolution of cell population functions by coupled differential equation systems rather than describing the dynamics at the level of individual cells [15, 19, 22, 23, 26–28, 54–56, 65–72]. The deterministic models presented here describe CML cell dynamics along the lines of the compartment concept proposed in Ref. [15], adding clonal competition [31], and using only one level of maturation/differentiation [69]. Stem cell mutation rate terms are included in the models in the straightforward, deterministic way suggested by Ref. [23].

Alternative deterministic models that take a clonal competition of normal and cancer cells into account have also been investigated [28, 69], linking other physiological phenomena to population dynamics, such as combination treatment [28] or the effects of immune response [23, 27].

Mathematical analysis of the test simulations performed with model A leads to the identification of two bifurcations of the stationary solutions and of three long term regimes, two stable line equilibria and one with unstable co-existence of two drug resistant clones (clones 2 and 3, cf. Sec. 3.2.2). The extensions of the *θ*_*s*2_ parameter space by *x*_*q*1_(0) and *ν*_*q*1_ in model B and by the feedback-control term for the activation of quiescent stem cells in model C do not introduce new bifurcations, because the *x*_*q*1_(*t*) reservoir will be exhausted in the limit *t* → ∞, reducing models B and C to model A asymptotically. Thus for the parameter regimes considered in the test simulations (cf. Secs. 3.2.1, 3.3 and 3.4.1), the three long term states (clone 2 or 3 alone or a clone 2 and 3 combination) can be realized by varying the single parameter *θ*_*s*2_ with all three equation systems. The deterministic dynamics of related approaches to CML cell evolution are investigated in more detail in Refs. [31, 34]; Ref. [33] covers in addition a stochastic ansatz.

From a methodological perspective, the present study contributes in particular with respect to two aspects:

- Our ansatz, notably model C, can be considered a modification of the deterministic approaches outlined in Refs. [15, 28, 31] in the sense that a quiescent stem cell reservoir *x*_*qi*_(*t*) is coupled to the cycling growth environment *x*_*si*_(*t*) in a new fashion.
- The analysis of the global dynamics of the stem cell compartment of model A in Sec. 3.2.2 demonstrates that information on the bifurcation landscape described by the solutions to such an equation system in a multidimensional parameter space can be systematically obtained based on the criteria derived in Refs. [33, 34]. Locating the branch points separating equilibria is important because the sensitivity of the solutions to parameter variations is reaching a maximum in their vicinity. The classification of the various equilibria and a comprehension of the dependence of the corresponding regions on bifurcation parameters is not only of interest for the interpretation of simulation results but also useful for predictions on how the biological system will react to therapy-induced modifications of CML clone rate constants *α*_*j*_ and *δ*_*j*_. This knowledge can be of value for the timing of CML treatment procedures.

The patient simulations outlined in Sec. 3.4.2 indicate that model C provides a satisfactory approximation to the complex clinical data of PP14, independent of the assumption whether a small clone 3 (V299_2) stem cell population pre-existed (Fig 10) or originated via mutations during *dasatinib* application (Fig 11). PP15 is a special case since our analysis provides evidence for the involvement of a third clone. Model C can describe the key features of PP15 within a two-clone framework based on the clinical datasets, but the accuracy of this approach is rather low (Fig 14, Table 5). However, the experimentally determined total leukemic burden suggests that a third clone, not explicitly identified in PP15, may play an important role, and the three-clone ansatz in fact elegantly yields an excellent match to the observed BCR-ABL1 values, again based on simulations implying an origin of clone 3 either preceding (Fig 12) or, by a mutation mechanism, within the *dasatinib* treatment interval (Fig 13). Interestingly, the best agreement between experiment and theory for PP15 can be reached by reducing model C to model B, i.e., deactivation of the *x*_*s*1_(*t*) population does not seem to play a major role in stem cell dynamics creating PP15, under the condition that a third clone *x*_*s*3_(*t*) is substantially involved.

The biological relevance of this investigation can be summarized as follows:

- Models of the type suggested in Eq (4) have in principle the flexibility to reproduce regrowth of population functions *x*_*si*_(*t*) and *x*_*di*_(*t*) by means of appropriately selected stem cell competition parameters *ω*_*ij*_, i.e., the inclusion of quiescent stem cell reservoirs *x*_*qi*_(*t*) is not required for this purpose. However, simulations of multiphasic decay patterns as observed for clone Y253H (*x*_*s*1_(*t*), *x*_*q*1_(*t*), *x*_*d*1_(*t*)) in PP14 (Figs 10 and 11) and, with a two-clone ansatz, also in PP15 (Fig 14) reveal a complex interplay of *x*_*q*1_(*t*) activation and *x*_*s*1_(*t*) deactivation rates. Solution of the fitting problem represented by PP15 is simplified via integration of an unconfirmed third clone, requiring only activation of *x*_*q*1_(*t*) to reach an accurate approximation to the clinical datasets, i.e., model B is sufficient in this case (Figs 12 and 13). The results underline that decline processes as experienced by strain Y253H in response to *dasatinib* administration could not be modelled without involvement of a quiescent growth environment.
- The question of whether substances like *imatinib*, *dasatinib* and *nilotinib* can target not only progenitor and differentiated CML cells but also CML stem cells in their bone marrow niches is a subject of intense research [7, 15, 23, 62, 73–75]. If a suppressing effect of a drug like *imatinib* on cycling wild-type CML stem cells is accepted, and if no resistant clones develop in a patient, then the next issue needs to be addressed: Can residual reservoirs of non-cycling wild-type CML stem cells be completely eliminated by time-limited *imatinib* administration [15]?
  The test and patient simulations of typical treatment profiles of CML patients with *imatinib*-resistant clones demonstrate that activation and deactivation of quiescent and cycling CML stem cells, respectively, are common phenomena in the course of TKI medication. The rapid and sustainable elimination of differentiated cell populations of clones like Y253H and F317L in PP7 (*dasatinib*) and PP35 (*nilotinib*), respectively, indicates that both agents have the potential to target active CML stem cells in their bone marrow niches and possibly eradicate both active and quiescent CML stem cells under certain circumstances.
  The simulations of PP14 and PP15 reported in this study are further compatible with the notion that quiescent stem cell pools (*x*_*q*1_(*t*)) of a clone like Y253H that is efficiently targeted by a CML drug both at the level of differentiated cells and of cycling stem cells are intensely activated to buffer the simultaneous decay of the *x*_*s*1_(*t*) and *x*_*d*1_(*t*) populations. As a consequence, the *x*_*q*1_(*t*) reservoirs drain rapidly and are eventually depleted. The net *x*_*q*1_(*t*) → *x*_*s*1_(*t*) transfer processes are accompanied by a retardation and finally acceleration of the decay of the *x*_*s*1_(*t*) and *x*_*d*1_(*t*) cell pools, leading to the observed multiphasic decline scenarios of the Y253H strain in PP14 and PP15.
  Our results support the viability of a complete extermination of cycling and non-cycling wild-type CML stem cells in patients by time-constrained *imatinib* therapy, assuming that the mechanism of buffering the decay of active stem cells followed by exhaustion of the quiescent stem cell pool in response to *imatinib* administration is realized in the same way as predicted for resistant clone Y253H. Under these conditions, *imatinib* treatment could represent a durable cure for CML if no resistance develops [25].
- The example of the patient simulations of PP15 (Sec. 3.4.2) shows that studies of this type can be helpful for the interpretation of patient data and for the design of treatment protocols. Our results point to the involvement of a third, *dasatinib*-resistant CML strain that acquires a differentiated cell BCR-ABL1 / GUS ratio of 1.0 × 10^−3^ only after ca. 190d of treatment (Figs 12 and 13).
- Simulations of PP14 (Figs 10 and 11) and PP15 (Figs 12 and 13) indicate that a distinction between pre-existence and a mutation origin (during *dasatinib* treatment) of stem cells of the respective clone 3 based on fits to sparse datasets of differentiated cell populations cannot be reliably made. Precise clinical information on stem cell populations is necessary to localize the appearance of a CML clone in time.

Future models will be able to implement ongoing experimental determination of key properties of CML and its treatment. In addition to the controversially discussed accessibility of stem cells to medication [7, 15, 21, 25, 28], key properties will include modelling of signalling systems more broadly to include effects of drug on individual components of the signalling pathways in CML, including e.g. off-target (i.e. non-ABL) effects of drug. These developments promise to enable knowledge based drug and treatment design to an unprecedented degree.
